# Development and Validation of a Natural Language Processing Tool to Generate the CONSORT Reporting Checklist for Randomized Clinical Trials

**DOI:** 10.1001/jamanetworkopen.2020.14661

**Published:** 2020-10-08

**Authors:** Fan Wang, Richard L. Schilsky, David Page, Robert M. Califf, Kei Cheung, Xiaofei Wang, Herbert Pang

**Affiliations:** 1 School of Public Health, Li Ka Shing Faculty of Medicine, The University of Hong Kong, Hong Kong, China; 2 American Society of Clinical Oncology, Alexandria, Virginia; 3 Department of Biostatistics and Bioinformatics, Duke University School of Medicine, Durham, North Carolina; 4 Duke Forge, Duke University School of Medicine, Durham, North Carolina; 5 Stanford University School of Medicine, Stanford, California; 6 Verily Life Sciences, South San Francisco, California; 7 Department of Emergency Medicine, Yale Center for Medical Informatics, Yale University School of Medicine, New Haven, Connecticut

## Abstract

**Importance:**

Adherence to the Consolidated Standards of Reporting Trials (CONSORT) for randomized clinical trials is associated with improvingquality because inadequate reporting in randomized clinical trials may complicate the interpretation and the application of findings to clinical care.

**Objective:**

To evaluate an automated reporting checklist generation tool that uses natural language processing (NLP), called CONSORT-NLP.

**Design, Setting, and Participants:**

This study used published journal articles as training, testing, and validation sets to develop, refine, and evaluate the CONSORT-NLP tool. Articles reporting randomized clinical trials were selected from 25 high-impact-factor journals under the following categories: (1) general and internal medicine, (2) oncology, and (3) cardiac and cardiovascular systems.

**Main Outcomes and Measures:**

For an evaluation of the performance of this tool, an accuracy metric defined as the number of correct assessments divided by all assessments was calculated.

**Results:**

The CONSORT-NLP tool uses the widely used Portable Document Format as an input file. Of the 37 CONSORT reporting items, 34 (92%) were included in the tool. Of these 34 reporting items, 30 were fully implemented; 28 (93%) of the fully implemented CONSORT reporting items had an accuracy of more than 90% for the validation set. The remaining 2 (7%) had an accuracy between 80% and 90% for the validation set. Two to 5 articles were selected from each of these journals for a total of 158 articles to establish a training set of 111 articles to train CONSORT-NLP for CONSORT reporting items, a testing set of 25 articles to refine CONSORT-NLP, and a validation set of 22 articles to assess the performance of CONSORT-NLP. The CONSORT-NLP tool used the Portable Document Format of the articles as input files. A CONSORT-NLP graphical user interface was built using Java in 2019. The time required to complete the CONSORT checklist manually vs using the CONSORT-NLP tool was compared for 30 articles. Two case studies for randomized clinical trials are provided as an illustration for the CONSORT-NLP tool. For the 30 articles investigated, CONSORT-NLP required a mean (SD) 23.0 (4.1) seconds, whereas the manual reviewer required a mean (SD) 11.9 (2.2), 22.6 (4.6), and 57.6 (7.1) minutes, for 3 reviewers, respectively.

**Conclusions and Relevance:**

The CONSORT-NLP tool is designed to assist in the reporting of randomized clinical trials. Potential users of CONSORT-NLP include clinicians, researchers, and scientists who plan to publish a randomized trial study in a peer-reviewed journal. The use of CONSORT-NLP may help them save substantial time when generating the CONSORT checklist. This tool may also be useful for manuscript reviewers and journal editors who review these articles.

## Key Points

**Question:**

Can natural language processing tools generate a Consolidated Standards of Reporting Trials (CONSORT) reporting checklist automatically for manuscripts of randomized clinical trials?

**Findings:**

An automated reporting checklist generation tool using natural language processing, CONSORT-NLP, was developed using 158 articles reporting randomized clinical trials; CONSORT-NLP performed well in the validation set evaluation of fully implemented reporting items (28 of 30 items [93%] achieved >90% accuracy, and the remaining 2 of 30 [7%] achieved between 80% and 90% accuracy) and requires on average 23 seconds to complete (human: 11.9-57.6 minutes).

**Meaning:**

Authors who plan to publish a randomized clinical trial with the CONSORT checklist may save substantial time by using CONSORT-NLP because this tool is an aid in completing the CONSORT checklist.

## Teaser

This study evaluates an automated reporting checklist generation tool that uses natural language processing to improve adherence to the Consolidated Standards of Reporting Trials (CONSORT) reporting checklist for randomized clinical trials (RCTs).

## Introduction

Inadequate reporting of randomized clinical trials (RCTs) may complicate the interpretation of study results and thereby negatively impact future research or even the application of study results to clinical care. Proper reporting may also improve the likelihood that published results are reproducible and may contribute to the transparency and structured thinking necessary for high-quality research. Enhancing and harmonizing reporting in publications may advance research and may ultimately improve patient outcomes.^1^ The well-known reporting guideline for RCTs is called the Consolidated Standards of Reporting Trials (CONSORT), and it has been a fundamental element of quality considerations in clinical trial reporting. At present, the CONSORT reporting guideline (updated in 2010) has already been cited more than 10 000 times.^2^

To improve adherence to the CONSORT reporting guideline, extensions of the guideline have been proposed.^3,4^ Despite efforts to promote proper reporting, adherence to the CONSORT reporting guideline remains an issue.^5,6^ For example, Limb et al^7^ reported that 63% of general surgical studies investigated did not report the funding sources. A recent literature search with the key words “randomized” AND “controlled” AND “trial” on PubMed yielded more than 40 000 articles in 2017. With these many articles, the issue regarding adherence can be significant. In addition, tremendous effort and resources are required to check these reporting items manually.

The structured format of CONSORT would seem to lend itself to natural language processing (NLP), thus enabling researchers to reduce that amount of effort and to ensure their own adherence but also providing a means for others to acquire the needed structured data in an automated fashion. Natural language processing is a subfield of artificial intelligence. In recent years, automated tools like NLP have increasingly been used in various biomedical research fields, such as oncology, dermatology, gastroenterology, neurology.^8,9,10,11,12^ The goal of this study is to develop an NLP tool for authors of RCTs to automatically generate a CONSORT report and check for CONSORT adherence. The latest version of CONSORT-NLP and a step-by-step tutorial guide (eAppendix in the Supplement) can be downloaded from http://www.consort-nlp.org.^13^

## Methods

### NLP Techniques in CONSORT-NLP

The CONSORT-NLP tool uses processing techniques that can be broadly categorized into syntactic and semantic approaches. Syntax-based NLP techniques focus on the grammatical structure of sentences. In CONSORT-NLP, we used syntax-based methods such as tokenization, lemmatization, part-of-speech tagging, sentence breaking, terminology extraction, and word segmentation.^14^ Lemmatization removes inflectional endings to return the canonical form of a word.

Semantics-based NLP techniques encompass the meaning of sentences. Among these techniques, CONSORT-NLP uses named entity recognition, relationship extraction, and word-sense disambiguation. Named entity recognition locates and classifies the name of real-world objects, such as WHO (ie, World Health Organization) for organizations and New York for locations. Relationship extraction identifies associations among named entities. Word-sense disambiguation recognizes the meaning of a word in a sentence where more than 1 meaning is possible. Our tool is primarily rule-based with the incorporation of previously machine learning–trained corpus. The study was performed from February 7, 2019, and January 31, 2020.

### Flow Diagram for CONSORT-NLP

**Figure 1.**
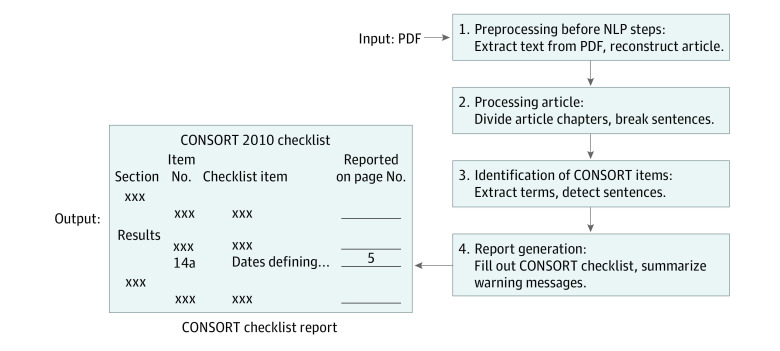
Simplified Flow Diagram of Consolidated Standards of Reporting Trials–Natural Language Processing (CONSORT-NLP) The 4-step flow process of CONSORT-NLP from PDF as input to CONSORT checklist generation.

The process of CONSORT-NLP mainly includes 4 steps (Figure 1). The first step involves the extraction of text from the PDF input before the reconstructed article is processed with NLP. Processing of the article begins in the second step. In brief, the reconstructed article is divided by sections and then further broken down into sentences. The third step involves the identification of CONSORT items. In this step, a combination of NLP methods is used to identify the relevant sentences for each CONSORT item. Item numbers 2a and 22 of CONSORT are less objective in nature, and item number 17b is only a recommendation. Therefore, they are not implemented in CONSORT-NLP. The final step is report generation. The CONSORT-NLP tool automatically fills out the CONSORT checklist in word document format and summarizes the notification messages, if applicable, in a separate word document.

### Graphical User Interface of CONSORT-NLP

**Figure 2.**
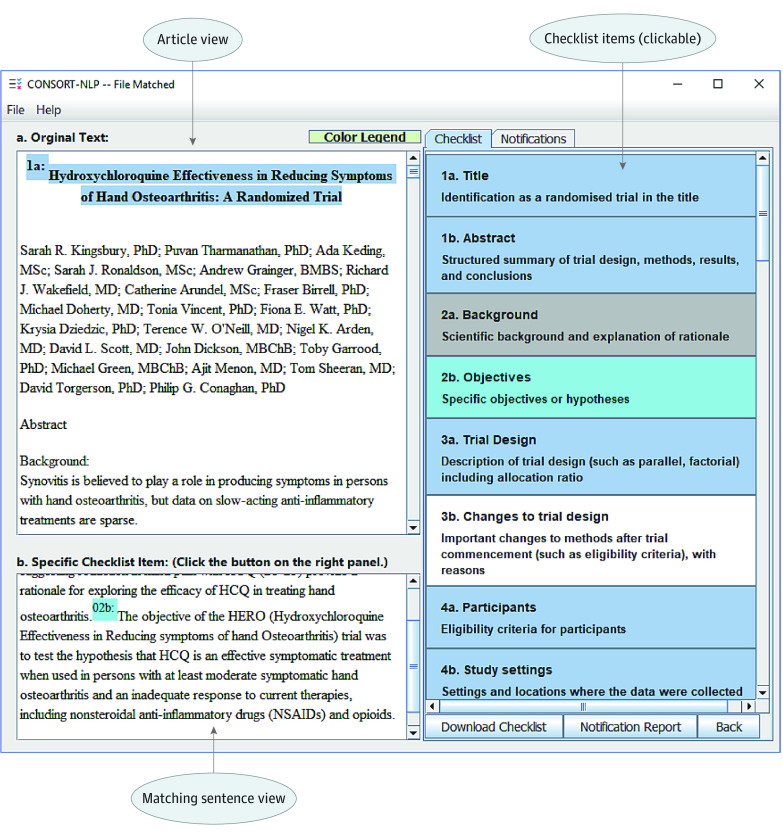
Case Study 1—Screenshot 1 A screenshot displaying the 3 main panels of Consolidated Standards of Reporting Trials–natural language processing (CONSORT-NLP): (1) Article view, (2) Matching sentence view, and (3) Checklist items.

The CONSORT-NLP tool has 3 main functions: article upload, feedback on CONSORT items, and report generation. There are 2 main pages of the graphical user interface. On page 1, we provide the upload page for the document to be checked by CONSORT-NLP. The second page consists of an interactive page where users are provided feedback on the CONSORT items. As displayed in Figure 2, the interactive page is divided into 3 parts: Article view, Matching sentence view, and Checklist items. Article view displays the text that highlights the relevant sentences of the checklist items. The buttons with different colors in checklist items are clickable. The blue highlighted text indicates that the sentence matches the checklist item. White text suggests that no relevant information in the article has been identified for the item. When a notification message is provided for the item, pink highlighting will be shown. For example, a notification message will be shown when the reporting item is presented in a different section of the manuscript from the one specified in the CONSORT checklist. Gray highlighting indicates that the item is not implemented in CONSORT-NLP. After a click of the button, the detected sentence(s) for that item are displayed in the matching sentence view. On page 2, a CONSORT report based on the reporting guideline^15^ can be generated using the tool as well. A summary of items with notification messages can also be exported. We present 2 real articles, one for randomized study on drug efficacy and another on comparing therapeutic options as case studies in the results section.

### Target Users

The CONSORT-NLP tool was designed for checks of CONSORT items in an RCT. Potential users of CONSORT-NLP include researchers, scientists, and clinicians who plan to publish an RCT in a peer-reviewed journal. This tool can also be useful for manuscript reviewers and journal editors who review these articles.

### Identification of Journals and Articles for Training, Testing, and Validating CONSORT-NLP

We selected journals from 3 major categories of medical research: (1) general and internal medicine, (2) oncology, and (3) cardiac and cardiovascular systems, according to their International Scientific Indexing impact factor. These journals are ranked in the top 50 in those categories and publish primarily original research. Articles reported with key phrases (ie, “randomized trial,” “randomised trial,” “randomized study,” or “randomised study”) in the title or subtitle were identified. Comments, reviews, meta-analyses, and studies focusing on additional analysis were excluded. We picked 2 to 5 articles from each of these chosen journals for a total of 158 articles to establish a training set of 111 articles to train CONSORT-NLP for CONSORT reporting items, a testing set of 25 articles to refine CONSORT-NLP, and a validation set of 22 articles to assess the performance of CONSORT-NLP.

### Metric for Assessing Performance

Accuracy is measured for each item across all articles. Correct assessment for a reporting item of an article means that (1) CONSORT-NLP correctly extracts the relevant reporting item from the article or (2) CONSORT-NLP reports null finding and the article truly does not contain the relevant reporting item. As is standard, accuracy for each item is measured as the number of articles correctly assessed divided by the total number of articles. Item number 10 of CONSORT was not fully implemented because of some ambiguity in the definition and so is not included in our accuracy calculation. Item numbers 13a, 13b, and 16 are also excluded from the accuracy calculation because some of the required information may be embedded in figures or tables. The remaining 30 implemented items were categorized into those achieving less 80% accuracy, 80% to 90% accuracy, and more than 90% accuracy in bar charts.

### Training CONSORT-NLP

During review of the articles in the training set, sentences describing CONSORT reporting items were manually extracted and summarized separately by item. We analyzed the sentences of each CONSORT item and developed a natural language processing strategy based on several common features in those sentences. We used these strategies to detect information from the training set articles. We filtered the extracted sentences into 3 groups: positive, negative, and undetected. The positive group includes sentences that are extracted with the relevant information about the CONSORT item. The negative group includes sentences that are extracted with information unrelated to the CONSORT item. Another group of sentences that contains the relevant information but is not detected by CONSORT-NLP is classified as undetected. We analyzed the sentences in the negative group and the undetected group to improve our strategies until the proportion of these 2 groups is less than 10% for the training set.

### Testing and Refining CONSORT-NLP

We used the testing set to pilot the first version of CONSORT-NLP. We checked the articles manually to assess whether the appropriate items were correctly detected. After reviewing all the articles, we calculated the accuracy of the first test version of CONSORT-NLP with the testing set. To refine this version, item search strategies were modified. The second test version of CONSORT-NLP resulted from the refinement.

### Validation of CONSORT-NLP

An independent set of articles in the validation set was used to evaluate the performance of the CONSORT-NLP. F.W. and H.P. independently reviewed the CONSORT-NLP output and the original article. A full listing of the articles used is provided in the eTable in the Supplement.

### Data Extraction and Timing

The CONSORT items and their descriptions (available from the CONSORT website at http://www.consort-statement.org)^16^ were studied. F.W. performed a detailed review on the implemented CONSORT items for each article and met with H.P. to discuss and resolve difficult reported item cases. To understand the difference in the time required to complete the CONSORT checklist manually vs the CONSORT-NLP tool, we timed the number of seconds or minute required to complete the CONSORT checklist for 30 articles manually. Ten articles each were randomly selected and assigned to F.W., X.W., and H.P. X.W. was added to represent an experienced reviewer but with less training on the CONSORT checklist. We compared the time required manually against the time needed by the CONSORT-NLP tool. CONSORT-NLP was developed with JAVA 8 Update 181, Python 3.5, and Stanford NER.^17^

## Results

### Characteristics of Included Articles

A total of 158 articles were included in this study. The study articles were obtained from 25 journals with an International Scientific Indexing impact factor (as of 2018) of 3 to 75 from 3 major categories of medical research: (1) general and internal medicine, (2) oncology, and (3) cardiac and cardiovascular systems. For general and internal medicine, 8 journals were selected, including the *New England Journal of Medicine*, *Lancet*, and *JAMA*. For oncology, 9 journals were selected, including *Lancet Oncology*, *Journal of Clinical Oncology*, and *Annals of Oncology*. For cardiac and cardiovascular systems, 8 journals were selected, including *Circulation*, *Journal of the American College of Cardiology*, and *Circulation Research*. Of the 158 articles, 111 (70%) were selected as the training set to allow us to train CONSORT-NLP for CONSORT reporting items. To improve CONSORT-NLP, 1 article from each selected journal was chosen for the testing set. Of all 159 articles, 22 (14%) were used to validate the performance of the tool, and 134 (85%) were published between March 2010 and July 2019 after the release of the CONSORT 2010 Statement. The Table presents the journals included and describes the characteristics of the training, testing, and validation sets.

**Table.** Characteristics of Included Articles

Journal	Impact factor (as of 2018)	No. (%) of articles		
		Training	Testing	Validation
General medicine				
*N Engl J Med*	70.7	5 (13)	1 (13)	1 (13)
*Lancet*	59.1	5 (13)	1 (13)	1 (13)
*JAMA*	51.3	5 (13)	1 (13)	1 (13)
*British Medical Journal*	27.6	4 (11)	1 (13)	1 (13)
*Annals of Internal Medicine*	19.3	5 (13)	1 (13)	1 (13)
*Canadian Medical Association Journal*	6.9	6 (16)	1 (13)	1 (13)
*Palliative Medicine*	5.0	3 (8)	1 (13)	1 (13)
*American Journal of Medicine*	4.8	5 (13)	1 (13)	1 (13)
Total No. (8 journals)		38	8	8
Oncology				
*Lancet Oncology*	35.4	5 (12)	1 (11)	1 (13)
*Journal of Clinical Oncology*	28.2	5 (12)	1 (11)	1 (13)
*Annals of Oncology*	14.2	5 (12)	1 (11)	1 (13)
*Journal of the National Cancer Institute*	10.2	5 (12)	1 (11)	1 (13)
*Journal of Hematology & Oncology*	8.7	2 (5)	1 (11)	1 (13)
*European Journal of Cancer*	6.7	5 (12)	1 (11)	1 (13)
*Cancer*	6.1	5 (12)	1 (11)	0 (0)
*British Journal of Cancer*	5.4	4 (10)	1 (11)	1 (13)
*International Journal of Cancer*	5.0	5 (12)	1 (11)	1 (13)
Total No. (9 journals)		41	9	8
Cardiology				
*European Heart Journal*	23.2	5 (15)	1 (13)	1 (17)
*Circulation*	23.1	4 (12)	1 (13)	1 (17)
*JACC*	18.6	5 (15)	1 (13)	1 (17)
*Circulation Research*	15.9	2 (6)	1 (13)	0 (0)
*JACC: Cardiovascular Intervention*	9.5	5 (15)	1 (13)	1 (17)
*Circulation: Cardiovascular Interventions*	6.1	3 (9)	1 (13)	0 (0)
*Heart*	5.0	5 (15)	1 (13)	1 (17)
*International Journal of Cardiology*	3.5	4 (12)	1 (13)	1 (17)
Total No. (8 journals)		33	8	6
Total No. (25 journals)		112	25	22

Abbreviations: *JACC*, *Journal of the American College of Cardiology*; *JAMA*, *Journal of the American Medical Association*; *N Engl J Med*, *New England Journal of Medicine*.

### Performance of CONSORT-NLP

**Figure 3.**
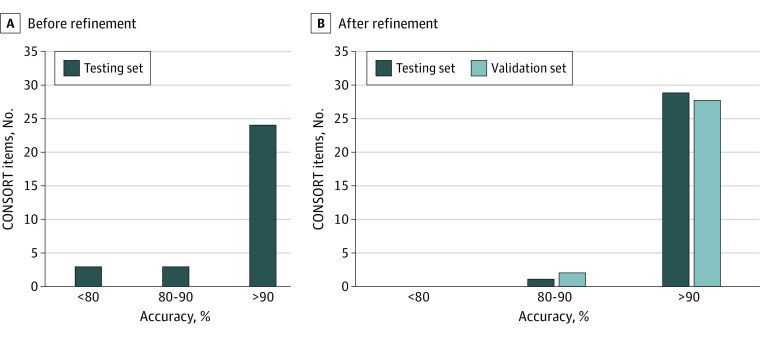
Performance of Consolidated Standards of Reporting Trials–Natural Language Processing (CONSORT-NLP) Compared with the tool before refinement, the updated CONSORT-NLP was able to achieve an accuracy of more than 90% for all items except for 1 item in the validation set. Accuracy is calculated as the number of articles correctly assessed divided by the total number of articles. Correct assessment for a reporting item of an article means that (1) CONSORT-NLP correctly extracts the relevant reporting item from the article or (2) CONSORT-NLP reports null finding and the article truly does not contain the relevant reporting item.

The accepted document format for CONSORT-NLP is the widely used PDF. Of the 37 CONSORT reporting items, 34 (92%) were included in our tool. A testing set of articles not included in the training set was used to evaluate the performance of the tool. Of the 30 fully implemented items, 24 (80%) had an accuracy of more than 90% (Figure 3). To further refine and enhance CONSORT-NLP, articles in the testing set were used for a new round of training. The updated CONSORT-NLP after refinement was able to achieve an accuracy of more than 90% for all items except for number 9 on allocation concealment mechanism for randomization (Figure 3). An independent validation set was used to assess the performance of the updated tool. Of the 30 fully implemented items, 28 (93%) had an accuracy of more than 90% (Figure 3).

The back-end code of CONSORT-NLP was written in Python. The graphical user interface was implemented with Java and was designed to run on various operating systems, including Windows, Mac, and Linux. On Windows 10 with Intel Core i7 3.40-GHz CPU, the mean run time of the program was approximately 12 seconds after the target article has been loaded in the tool. For the 30 articles investigated, CONSORT-NLP required a mean (SD) 23.0 (4.1) seconds, whereas the reviewers F.W., H.P., and X.W. required a mean (SD) 11.9 (2.2), 22.6 (4.6), and 57.6 (7.1) minutes, respectively.

#### Case Study 1

The first case study is an example of an RCT for testing drug efficacy and safety. The primary objective of the HERO (Hydroxychloroquine Effectiveness in Reducing Symptoms of hand Osteoarthritis) trial is to determine the effectiveness of hydroxychloroquine vs placebo as a symptomatic treatment for hand osteoarthritis.^18^ We used CONSORT-NLP to generate the CONSORT 2010 checklist.^15^

**Figure 4.**
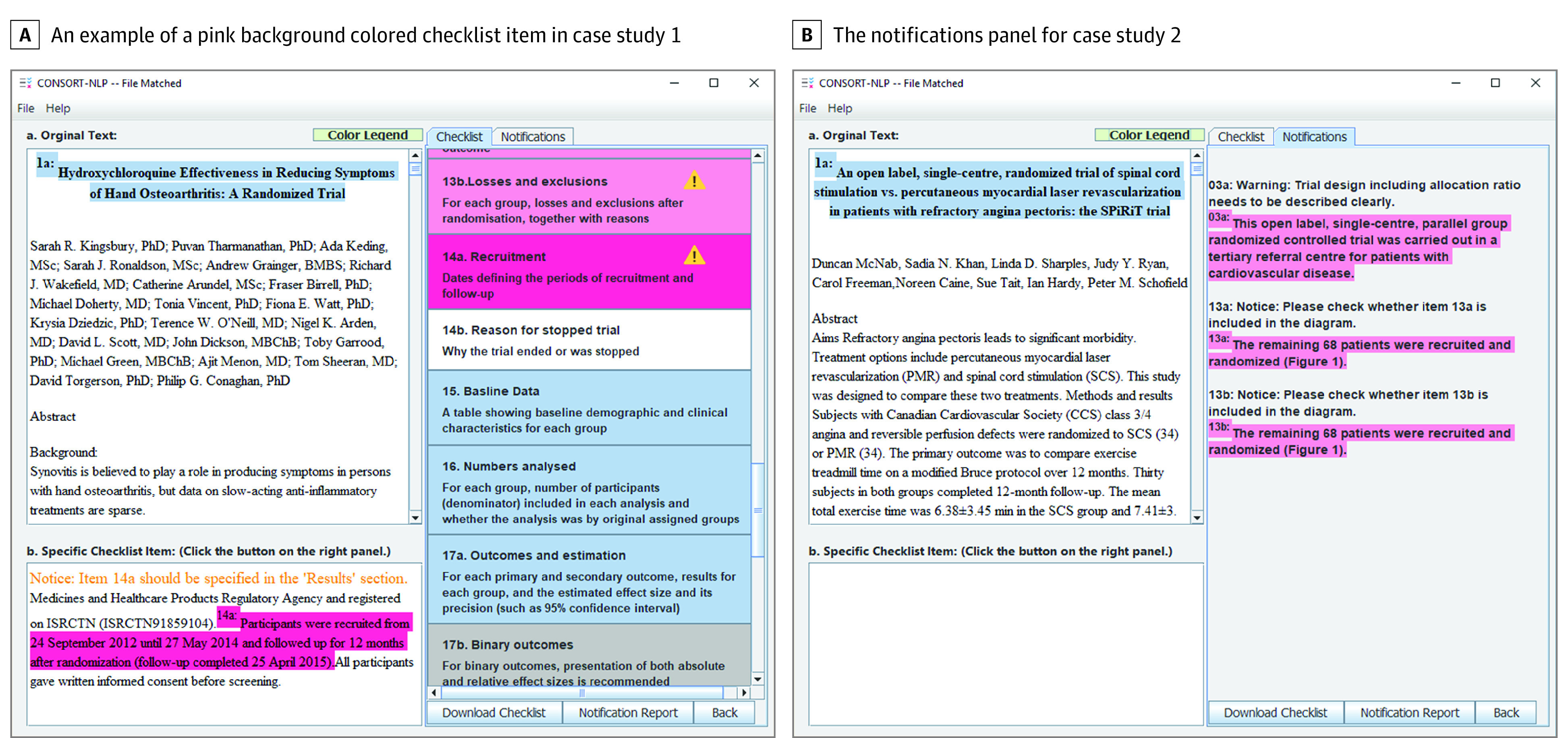
Screenshots for Case Study 1 and Case Study 2

On page 1 (eAppendix in the Supplement), we uploaded the HERO article to CONSORT-NLP in PDF format. After approximately 10 seconds of wait time, CONSORT-NLP will refresh to the second page as shown in Figure 2. The second page provides feedback on the CONSORT items. In Figure 2, the sentence highlighted in blue in the Article view with prefix “1a” indicates that the sentence was matched to item 1a: Identification as a randomized trial in the title. The buttons with different colors in the Checklist items are clickable. In Figure 2, we demonstrate the result after clicking the light blue–highlighted item 2b: Specific objectives or hypotheses. After clicking, the detected sentence for the objective of HERO trial is displayed in the Matching sentence view. In Figure 4A, a pink-highlighted item was clicked; it indicates that a notification message is available for this item. As shown in Matching sentence view, we observe that CONSORT-NLP found a sentence that matches this item; however, this sentence should be present in the Results section of an article instead of the current Method section according to the CONSORT guideline. On page 2, a CONSORT report based on the reporting guideline is generated using our tool as well (eAppendix in the Supplement). Messages in the Notifications panel can also be exported (eAppendix in the Supplement).

#### Case Study 2

The second case study is an example of an RCT for comparing the effect of 2 therapeutic options. The primary objective of the SPiRiT trial is to compare the effect of percutaneous myocardial revascularization (PMR) and spinal cord stimulation (SCS) on treadmill exercise time among patients with refractory angina pectoris.^19^ We used CONSORT-NLP to generate the CONSORT 2010 checklist.^15^

On page 1, we uploaded the SPiRiT article to CONSORT-NLP in PDF format. After approximately 11 seconds of wait time, CONSORT-NLP will refresh to the second page, similar to Figure 2 of case study 1. In Figure 4B, the result after clicking the “Notifications” tab is shown. After clicking on it, the Notifications panel is now displayed instead of the Checklist items panel. The Notifications panel summarizes the items for the user to take note for the article. The CONSORT-NLP tool now shows 3 notification messages for this article. In the first notification message, CONSORT-NLP finds a sentence that describes the trial design; however, the allocation ratio is not found. The second and third notification messages remind the user to check whether Figure 1 contains the information for items 13a and 13b.

## Discussion

The CONSORT-NLP tool may help improve the quality of reporting of RCTs and the efficiency in generating the CONSORT reporting checklist by enabling authors and reviewers to automatically assess how well their manuscripts conform to the CONSORT reporting standard. The CONSORT checklist is also provided by this tool and can be submitted along with the manuscript for journal publication. Guidelines like the CONSORT are essential to promote the reproducibility and interpretability of RCTs.

In recent years, tools have been developed to help authors follow the CONSORT guidelines. For example, web-based writing aid tools based on the CONSORT statement have been developed and studied.^20,21^ One of them contains a series of textboxes for authors to fill out that address CONSORT items. The other tool has been implemented at the manuscript revision stage. Neither of these tools is based on NLP, nor do they automatically check for compliance of a draft manuscript directly. Although there are reported improvements since the introduction of the CONSORT guidelines and new tools, calls have been made for better adherence to the standards, even in high-impact general medical journals.^22^ The situation is particularly severe in specialty journals for disciplines such as anesthesiology, cardiology, psychiatry, and surgery.^5,23,24,25^ Adherence to guidelines is associated with the impact factors and is less satisfactory in lower-impact-factor journals than higher ones because high-impact-factor journals are, in general, more resourceful.^26^ Therefore, we believe that CONSORT-NLP is timely and may help readers, publishers, and authors of RCTs in the biomedical research ecosystem.

### Limitations

This study is not without limitations. Item numbers 2a, 22, and 17b of the 37 CONSORT items have not been implemented owing to their complexity and the level of subjectivity involved in assessing adherence to them. However, with more data and more sophisticated training algorithms, the CONSORT-NLP tool may be improved further.

## Conclusions

Currently, for the majority of journals, the responsibility for adhering to the reporting criteria in the CONSORT guidelines still lies with the author. The CONSORT-NLP tool, a standalone software tested on the major desktop operating systems, takes advantage of NLP techniques to automatically generate a report on the ready-to-submit manuscript. Moreover, an editable CONSORT checklist can also be generated for the manuscript. This time-saving feature may encourage adoption of the tool. The CONSORT-NLP tool may improve the reporting and interpretability of RCTs. The tool is not intended to completely replace human evaluation of CONSORT adherence but to encourage and increase the adherence to CONSORT guidelines by reducing the time for completing checklists manually.

## Notes

Supplement. **eTable.** Supplementary List **eAppendix.** Step-by-Step User Guide
