# Supplementary material for: Development and Validation of a Natural Language Processing Tool to Generate the CONSORT Reporting Checklist for Randomized Clinical Trials
Source: JAMA Netw Open. 2020 Oct 8;3(10):e2014661. doi: 10.1001/jamanetworkopen.2020.14661 (PMC7545295; doi:10.1001/jamanetworkopen.2020.14661)
Supplement: Supplement. — eTable. Supplementary List eAppendix. Step-by-Step User Guide [file jamanetwopen-e2014661-s001.pdf]

Supplementary Online Content

Wang F, Schilsky RL, Page D, et al. Development and validation of a natural language processing tool to generate the CONSORT reporting checklist for randomized clinical trials. *JAMA Netw Open*. 2020;3(10):e2014661. doi:10.1001/jamanetworkopen.2020.14661

eTable 1. Supplementary List

eAppendix. Step-by-Step User Guide

This supplementary material has been provided by the authors to give readers additional information about their work.

© 2020 Wang F et al. *JAMA Network Open.*

eTable 1. Supplementary List

Medicine, general & internal

	Authors	Article Title	Journal
1	Lucas et al. (2011)	Hand-carried echocardiography by hospitalists: a randomized trial	American Journal of Medicine
2	Gariballa et al. (2006)	A randomized, double-blind, placebo-controlled trial of nutritional supplementation during acute illness	American Journal of Medicine
3	Reid et al. (2006)	Randomized controlled trial of calcium in healthy older women	American Journal of Medicine
4	Ng et al. (2015)	Nutritional, physical, cognitive, and combination interventions and frailty reversal among older adults: a randomized controlled trial	American Journal of Medicine
5	Patkar et al. (2007)	A randomized, controlled, trial of controlled release paroxetine in fibromyalgia	American Journal of Medicine
6	Fonseca et al. (2013)	Metanx in type 2 diabetes with peripheral neuropathy: a randomized trial	American Journal of Medicine
7	Jaiswal et al. (2018)	Melatonin and sleep in preventing hospitalized delirium: a randomized clinical trial	American Journal of Medicine
8	Roerink et al. (2017)	Cytokine inhibition in patients with chronic fatigue syndrome: a randomized trial	Annals of Internal Medicine
9	Vassy et al. (2017)	The impact of whole-genome sequencing on the primary care and outcomes of healthy adult patients: a pilot randomized trial	Annals of Internal Medicine
10	Goebel et al. (2017)	Low-dose intravenous immunoglobulin treatment for long-standing complex regional pain syndrome: a randomized trial	Annals of Internal Medicine
11	Kingsbury et al. (2018)	Hydroxychloroquine effectiveness in reducing symptoms of hand osteoarthritis: a randomized trial	Annals of Internal Medicine
12	Sulkowski et al. (2013)	Combination therapy with telaprevir for chronic hepatitis C virus genotype 1 infection in patients with HIV: a randomized trial	Annals of Internal Medicine
13	Guldvog et al. (2019)	Thyroidectomy versus medical management for euthyroid patients with hashimoto disease and persisting symptoms: a randomized trial	Annals of Internal Medicine
14	Beck et al. (2017)	Continuous glucose monitoring versus usual care in patients with type 2 diabetes receiving multiple daily insulin injections: a randomized trial	Annals of Internal Medicine
15	Jull et al. (2017)	Low dose aspirin as adjuvant treatment for venous leg ulceration: pragmatic, randomised, double blind, placebo controlled trial (Aspirin4VLU)	British Medical Journal
16	Hess et al. (2016)	Shared decision making in patients with low risk chest pain: prospective randomized pragmatic trial	British Medical Journal
17	Boden et al. (2018)	Preoperative physiotherapy for the prevention of respiratory complications after upper abdominal surgery: pragmatic, double blinded, multicentre randomised controlled trial	British Medical Journal
18	DREAMS et al. (2017)	Dexamethasone versus standard treatment for postoperative nausea and vomiting in gastrointestinal surgery: randomised controlled trial (DREAMS Trial)	British Medical Journal
19	Wang et al. (2018)	Effect of tai chi versus aerobic exercise for fibromyalgia: comparative effectiveness randomized controlled trial	British Medical Journal
20	Abt et al. (2018)	Comparison of prostatic artery embolisation (PAE) versus transurethral resection of the prostate (TURP) for benign prostatic hyperplasia: randomised, open label, non-inferiority trial	British Medical Journal
21	Usichenko et al. (2007)	Auricular acupuncture for pain relief after ambulatory knee surgery: a randomized trial	Canadian Medical Association Journal
22	Reid et al. (2014)	Effect of an intervention to improve the cardiovascular health of family members of patients with coronary artery disease: a randomized trial	Canadian Medical Association Journal
23	Poonai et al. (2014)	Oral administration of morphine versus ibuprofen to manage postfracture pain in children: a randomized trial	Canadian Medical Association Journal
24	Grey et al. (2017)	Duration of antiresorptive activity of zoledronate in postmenopausal women with osteopenia: a randomized, controlled multidose trial	Canadian Medical Association Journal
25	Cole et al. (2006)	Systematic detection and multidisciplinary care of depression in older medical inpatients: a randomized trial	Canadian Medical Association Journal
26	Agarwal et al. (2018)	Evaluation of a community paramedicine health promotion and lifestyle risk assessment program for older adults who live in social housing: a cluster randomized trial	Canadian Medical Association Journal
27	Babio et al. (2014)	Mediterranean diets and metabolic syndrome status in the PREDIMED randomized trial	Canadian Medical Association Journal
28	Bennett-Guerrero et al. (2010)	Effect of an implantable gentamicin-collagen sponge on sternal wound infections following cardiac surgery: a randomized trial	Journal of the American Medical Association
29	Stergiopoulos et al. (2015)	Effect of scattered-site housing using rent supplements and intensive case management on housing stability among homeless adults with mental illness: a randomized trial	Journal of the American Medical Association
30	Sulkowski et al. (2015)	Ombitasvir, paritaprevir co-dosed with ritonavir, dasabuvir, and ribavirin for hepatitis C in patients co-infected with HIV-1: a randomized trial	Journal of the American Medical Association
31	Engel et al. (2012)	Early surgical therapy for drug-resistant temporal lobe epilepsy: a randomized trial	Journal of the American Medical Association
32	Dysken et al. (2014)	Effect of vitamin E and memantine on functional decline in Alzheimer disease: the TEAM-AD VA cooperative randomized trial	Journal of the American Medical Association
33	Räber et al. (2012)	Effect of biolimus-eluting stents with biodegradable polymer vs bare-metal stents on cardiovascular events among patients with acute myocardial infarction: the COMFORTABLE AMI randomized trial	Journal of the American Medical Association
34	Kawazoe et al. (2017)	Effect of dexmedetomidine on mortality and ventilator-free days in patients requiring mechanical ventilation with sepsis: a randomized clinical trial	Journal of the American Medical Association
35	Heinemann et al. (2018)	Real-time continuous glucose monitoring in adults with type 1 diabetes and impaired hypoglycaemia awareness or severe hypoglycaemia treated with multiple daily insulin injections (HypoDE): a multicentre, randomised controlled trial	Lancet
36	Harrison et al. (2018)	NGM282 for treatment of non-alcoholic steatohepatitis: a multicentre, randomised, double-blind, placebo-controlled, phase 2 trial	Lancet
37	Kaufmann et al. (2018)	Mesh versus suture repair of umbilical hernia in adults: a randomised, double-blind, controlled, multicentre trial	Lancet
38	Decramer et al. (2005)	Effects of N-acetylcysteine on outcomes in chronic obstructive pulmonary disease (Bronchitis Randomized on NAC Cost-Utility Study, BRONCUS): a randomised placebo-controlled trial	Lancet
39	Jacobs et al. (2016)	Ovarian cancer screening and mortality in the UK Collaborative Trial of Ovarian Cancer Screening (UKCTOCS): a randomised controlled trial	Lancet
40	Rodgers et al. (2019)	Robot assisted training for the upper limb after stroke (RATULS): a multicentre randomised controlled trial	Lancet
41	van et al. (2018)	Personalised perioperative care by e-health after intermediate-grade abdominal surgery: a multicentre, single-blind, randomised, placebo-controlled trial	Lancet
42	Ba et al. (2015)	A randomized trial of icatibant in ACE-inhibitor-induced angioedema	New England Journal of Medicine
43	Buchbinder et al. (2009)	A randomized trial of vertebroplasty for painful osteoporotic vertebral fractures	New England Journal of Medicine
44	Barrett et al. (2013)	A randomized trial of planned cesarean or vaginal delivery for twin pregnancy	New England Journal of Medicine
45	Taggart et al. (2016)	Randomized trial of bilateral versus single internal-thoracic-artery grafts	New England Journal of Medicine
46	Wolfe et al. (2016)	Randomized trial of thymectomy in myasthenia gravis	New England Journal of Medicine
47	Hajek et al. (2019)	A randomized trial of E-cigarettes versus nicotine-replacement therapy	New England Journal of Medicine
48	Tuuli et al. (2016)	A randomized trial comparing skin antiseptic agents at cesarean delivery	New England Journal of Medicine
49	Groenvold et al. (2017)	Randomised clinical trial of early specialist palliative care plus standard care versus standard care alone in patients with advanced cancer: The Danish Palliative Care Trial	Palliative Medicine
50	Brazil et al. (2018)	Effectiveness of advance care planning with family carers in dementia nursing homes: A paired cluster randomized controlled trial	Palliative Medicine
51	Ahmedzai et al. (2012)	A randomized, double-blind, active-controlled, double-dummy, parallel-group study to determine the safety and efficacy of oxycodone/naloxone prolonged-release tablets in patients with moderate/severe, chronic cancer pain	Palliative Medicine
52	Nordly et al. (2019)	Systematic fast-track transition from oncological treatment to dyadic specialized palliative home care: DOMUS - a randomized clinical trial	Palliative Medicine
53	Pilegaard et al. (2018)	The 'Cancer Home-Life Intervention': A randomised controlled trial evaluating the efficacy of an occupational therapy-based intervention in people with advanced cancer	Palliative Medicine

© 2020 Wang F et al. *JAMA Network Open.*

Oncology

	Authors	Article Title	Journal
1	Ek et al. (2018)	Randomized phase III trial of low-molecular-weight heparin enoxaparin in addition to standard treatment in small-cell lung cancer: the RASTEN trial	Annals of Oncology
2	Chang et al. (2002)	A phase III randomized trial of 5-fluorouracil, doxorubicin, and mitomycin C versus 5-fluorouracil and mitomycin C versus 5-fluorouracil alone in curatively resected gastric cancer	Annals of Oncology
3	Ajani et al. (2013)	A phase II randomized trial of induction chemotherapy versus no induction chemotherapy followed by preoperative chemoradiation in patients with esophageal cancer	Annals of Oncology
4	Dear et al. (2012)	Impact of a cancer clinical trials web site on discussions about trial participation: a cluster randomized trial	Annals of Oncology
5	Coombes et al. (2005)	High dose chemotherapy and autologous stem cell transplantation as adjuvant therapy for primary breast cancer patients with four or more lymph nodes involved: long-term results of an international randomised trial	Annals of Oncology
6	Bajetta et al. (2014)	Randomized trial on adjuvant treatment with FOLFIRI followed by docetaxel and cisplatin versus 5-fluorouracil and folinic acid for radically resected gastric cancer	Annals of Oncology
7	Belani et al. (2017)	A randomized trial of TLR-2 agonist CADI-05 targeting desmocollin-3 for advanced non-small-cell lung cancer	Annals of Oncology
8	Hall et al. (2017)	A randomised phase II trial and feasibility study of palliative chemotherapy in frail or elderly patients with advanced gastroesophageal cancer (321GO)	British Journal of Cancer
9	Han et al. (2018)	Anlotinib as a third-line therapy in patients with refractory advanced non-small-cell lung cancer: a multicentre, randomised phase II trial (ALTER0302)	British Journal of Cancer
10	Sun et al. (2018)	Pazopanib maintenance after first-line etoposide and platinum chemotherapy in patients with extensive disease small-cell lung cancer: a multicentre, randomised, placebo-controlled Phase II study (KCSG-LU12-07)	British Journal of Cancer
11	Moss et al. (2005)	Randomised controlled trial of mammographic screening in women from age 40: predicted mortality based on surrogate outcome measures	British Journal of Cancer
12	Pegram et al. (2019)	PF-05280014 (a trastuzumab biosimilar) plus paclitaxel compared with reference trastuzumab plus paclitaxel for HER2-positive metastatic breast cancer: a randomised, double-blind study	British Journal of Cancer
13	Knödler et al. (2018)	Randomised phase II trial to investigate catumaxomab (anti-EpCAM × anti-CD3) for treatment of peritoneal carcinomatosis in patients with gastric cancer	British Journal of Cancer
14	Schover et al. (2012)	A randomized trial of internet-based versus traditional sexual counseling for couples after localized prostate cancer treatment	Cancer
15	Donnelly et al. (2010)	A randomized trial of external beam radiotherapy versus cryoablation in patients with localized prostate cancer	Cancer
16	Parker et al. (2013)	A multisite, community oncology-based randomized trial of a brief educational intervention to increase communication regarding complementary and alternative medicine	Cancer
17	Downs et al. (2008)	A prospective randomized trial of thalidomide with topotecan compared with topotecan alone in women with recurrent epithelial ovarian carcinoma	Cancer
18	Cianfrocca et al. (2010)	Randomized trial of paclitaxel versus pegylated liposomal doxorubicin for advanced human immunodeficiency virus-associated Kaposi sarcoma: evidence of symptom palliation from chemotherapy	Cancer
19	Sanoff et al. (2018)	Multicenter, randomized, double-blind phase 2 trial of FOLFIRI with regorafenib or placebo as second-line therapy for metastatic colorectal cancer	Cancer
20	Geerse et al. (2017)	Structural distress screening and supportive care for patients with lung cancer on systemic therapy: A randomised controlled trial	European Journal of Cancer
21	Ursu et al. (2017)	Intracerebral injection of CpG oligonucleotide for patients with de novo glioblastoma-A phase II multicentric, randomised study	European Journal of Cancer
22	Lindman et al. (2018)	A randomised study of tailored toxicity-based dosage of fluorouracil-epirubicin-cyclophosphamide chemotherapy for early breast cancer (SBG 2000-1)	European Journal of Cancer
23	Vergote et al. (2017)	A phase 2 randomised discontinuation trial of cabozantinib in patients with ovarian carcinoma	European Journal of Cancer
24	Mao et al. (2014)	A randomised trial of electro-acupuncture for arthralgia related to aromatase inhibitor use	European Journal of Cancer
25	Matsuda et al. (2018)	A randomised-controlled trial of 1-year adjuvant chemotherapy with oral tegafur-uracil versus surgery alone in stage II colon cancer: SACURA trial	European Journal of Cancer
26	Mukherjee et al. (2017)	NEOSCOPE: A randomised phase II study of induction chemotherapy followed by oxaliplatin/capecitabine or carboplatin/paclitaxel based pre-operative chemoradiation for resectable oesophageal adenocarcinoma	European Journal of Cancer
27	Schmidt et al. (2015)	Effects of resistance exercise on fatigue and quality of life in breast cancer patients undergoing adjuvant chemotherapy: a randomized controlled trial	International Journal of Cancer
28	Tainio et al. (2016)	Randomised trial on treatment of vaginal intraepithelial neoplasia-Imiquimod, laser vaporisation and expectant management	International Journal of Cancer
29	Bairati et al. (2006)	Antioxidant vitamins supplementation and mortality: a randomized trial in head and neck cancer patients	International Journal of Cancer
30	Nagao et al. (2015)	Treatment of oral leukoplakia with a low-dose of beta-carotene and vitamin C supplements: a randomized controlled trial	International Journal of Cancer
31	Meyer et al. (2008)	Interaction between antioxidant vitamin supplementation and cigarette smoking during radiation therapy in relation to long-term effects on recurrence and mortality: a randomized trial among head and neck cancer patients	International Journal of Cancer
32	Schmidt et al. (2014)	Effects of resistance exercise on fatigue and quality of life in breast cancer patients undergoing adjuvant chemotherapy: A randomized controlled trial	International Journal of Cancer
33	Han et al. (2017)	Combination of chemotherapy and gefitinib as first-line treatment for patients with advanced lung adenocarcinoma and sensitive EGFR mutations: A randomized controlled trial	International Journal of Cancer
34	Catton et al. (2017)	Randomized trial of a hypofractionated radiation regimen for the treatment of localized prostate cancer	Journal of Clinical Oncology
35	Arcangeli et al. (2017)	Moderate hypofractionation in high-risk, organ-confined prostate cancer: final results of a phase III randomized trial	Journal of Clinical Oncology
36	Butow et al. (2017)	Randomized trial of conquerFear: a novel, theoretically based psychosocial intervention for fear of cancer recurrence	Journal of Clinical Oncology
37	Brown et al. (2016)	Randomized trial of hepatic artery embolization for hepatocellular carcinoma using doxorubicin-eluting microspheres compared with embolization with microspheres alone	Journal of Clinical Oncology
38	Leighl et al. (2011)	Supporting treatment decision making in advanced cancer: a randomized trial of a decision aid for patients with advanced colorectal cancer considering chemotherapy	Journal of Clinical Oncology
39	Bairati et al. (2005)	Randomized trial of antioxidant vitamins to prevent acute adverse effects of radiation therapy in head and neck cancer patients	Journal of Clinical Oncology
40	Chan et al. (2018)	Analysis of Plasma Epstein-Barr Virus DNA in Nasopharyngeal Cancer After Chemoradiation to Identify High-Risk Patients for Adjuvant Chemotherapy: A Randomized Controlled Trial	Journal of Clinical Oncology
41	Wun et al. (2013)	A double-blind, randomized, multicenter phase 2 study of prasugrel versus placebo in adult patients with sickle cell disease	Journal of Hematology and Oncology
42	Li et al. (2017)	Platelet desialylation is a novel mechanism and a therapeutic target in thrombocytopenia during sepsis: an open-label, multicenter, randomized controlled trial	Journal of Hematology and Oncology
43	Wang et al. (2012)	A randomized, double-blind, placebo-controlled phase 2 study evaluating the efficacy and safety of romiplostim treatment of patients with low or intermediate-1 risk myelodysplastic syndrome receiving lenalidomide	Journal of Hematology and Oncology
44	Baron et al. (2015)	Non-myeloablative allogeneic hematopoietic cell transplantation following fludarabine plus 2 Gy TBI or ATG plus 8 Gy TLI: a phase II randomized study from the Belgian Hematological Society	Journal of Hematology and Oncology
45	Zachariae et al. (2018)	Internet-delivered cognitive-behavioral therapy for insomnia in breast cancer survivors: a randomized controlled trial	Journal of the National Cancer Institute
46	Ruers et al. (2017)	Local treatment of unresectable colorectal liver metastases: results of a randomized phase II trial	Journal of the National Cancer Institute
47	Lee et al. (2010)	Randomized trial of radiotherapy plus concurrent-adjuvant chemotherapy vs radiotherapy alone for regionally advanced nasopharyngeal carcinoma	Journal of the National Cancer Institute
48	Sali et al. (2015)	Reduced and full-preparation CT colonography, fecal immunochemical test, and colonoscopy for population screening of colorectal cancer: a randomized trial	Journal of the National Cancer Institute
49	Dearnaley et al. (2003)	A double-blind, placebo-controlled, randomized trial of oral sodium clodronate for metastatic prostate cancer (MRC PR05 Trial)	Journal of the National Cancer Institute
50	Kadan-Lottick et al. (2018)	Randomized trial of the impact of empowering childhood cancer survivors with survivorship care plans	Journal of the National Cancer Institute
51	Denis et al. (2017)	Randomized trial comparing a web-mediated follow-up with routine surveillance in lung cancer patients	Journal of the National Cancer Institute
52	Tang et al. (2018)	Concurrent chemoradiotherapy with nedaplatin versus cisplatin in stage II-IVB nasopharyngeal carcinoma: an open-label, non-inferiority, randomised phase 3 trial	Lancet Oncology
53	De et al. (2018)	Adjuvant anastrozole versus exemestane versus letrozole, upfront or after 2 years of tamoxifen, in endocrine-sensitive breast cancer (FATA-GIM3): a randomised, phase 3 trial	Lancet Oncology
54	Symonds et al. (2015)	Cediranib combined with carboplatin and paclitaxel in patients with metastatic or recurrent cervical cancer (CIRCCa): a randomised, double-blind, placebo-controlled phase 2 trial	Lancet Oncology
55	Walker et al. (2016)	Pretreatment with anti-thymocyte globulin versus no anti-thymocyte globulin in patients with haematological malignancies undergoing haemopoietic cell transplantation from unrelated donors: a randomised, controlled, open-label, phase 3, multicentre trial	Lancet Oncology
56	Shore et al. (2016)	Efficacy and safety of enzalutamide versus bicalutamide for patients with metastatic prostate cancer (TERRAIN): a randomised, double-blind, phase 2 study	Lancet Oncology
57	Hofvind et al. (2019)	Two-view digital breast tomosynthesis versus digital mammography in a population-based breast cancer screening programme (To-Be): a randomised, controlled trial	Lancet Oncology
58	Dreno et al. (2018)	MAGE-A3 immunotherapeutic as adjuvant therapy for patients with resected, MAGE-A3-positive, stage III melanoma (DERMA): a double-blind, randomised, placebo-controlled, phase 3 trial	Lancet Oncology

© 2020 Wang F et al. *JAMA Network Open.*

Cardiac & cardiovascular systems

	Authors	Article Title	Journal
1	Massie et al. (2009)	Randomized trial of warfarin, aspirin, and clopidogrel in patients with chronic heart failure: the Warfarin and Antiplatelet Therapy in Chronic Heart Failure (WATCH) trial	Circulation
2	Tsuyuki et al. (2015)	Randomized trial of the effect of pharmacist prescribing on improving blood pressure in the community: The Alberta Clinical Trial in Optimizing Hypertension (RxACTION)	Circulation
3	Geva et al. (2010)	Randomized trial of pulmonary valve replacement with and without right ventricular remodeling surgery	Circulation
4	Bergersen et al. (2011)	Randomized trial of cutting balloon compared with high-pressure angioplasty for the treatment of resistant pulmonary artery stenosis	Circulation
5	Lee et al. (2019)	Randomized Trial Evaluating Percutaneous Coronary Intervention for the Treatment of Chronic Total Occlusion	Circulation
6	Andreozzi et al. (2015)	Sulodexide for the prevention of recurrent venous thromboembolism: the sulodexide in secondary prevention of recurrent deep vein thrombosis (SURVET) study: a multicenter, randomized, double-blind, placebo-controlled trial	Circulation
7	Wang et al. (2015)	Cluster-randomized clinical trial examining the impact of platelet function testing on practice: the treatment with adenosine diphosphate receptor inhibitors: longitudinal assessment of treatment patterns and events after acute coronary syndrome prospective open label antiplatelet therapy study	Circulation - Cardiovascular interventions
8	Alazzoni et al. (2015)	Randomized controlled trial of radiation protection with a patient lead shield and a novel, nonlead surgical cap for operators performing coronary angiography or intervention	Circulation - Cardiovascular interventions
9	Russo et al. (2009)	A randomized controlled trial of angiography versus intravascular ultrasound-directed bare-metal coronary stent placement (the AVID Trial)	Circulation - Cardiovascular interventions
10	Rodés-Cabau et al. (2016)	Sealing intermediate nonobstructive coronary saphenous vein graft lesions with drug-eluting stents as a new approach to reducing cardiac events: a randomized controlled trial	Circulation - Cardiovascular interventions
11	Jones et al. (2015)	Randomized phase 2 trial of intracoronary nitrite during acute myocardial infarction	Circulation Research
12	Jimenez-Quevedo et al. (2014)	Selected CD133 progenitor cells to promote angiogenesis in patients with refractory angina: final results of the PROGENITOR randomized trial	Circulation Research
13	Jones et al. (2015)	Randomized phase 2 trial of intracoronary nitrite during acute myocardial infarction	Circulation Research
14	Ray et al. (2014)	The effect of cholesteryl ester transfer protein inhibition on lipids, lipoproteins, and markers of HDL function after an acute coronary syndrome: the dal-ACUTE randomized trial	European Heart Journal
15	Bermejo et al. (2018)	Sildenafil for improving outcomes in patients with corrected valvular heart disease and persistent pulmonary hypertension: a multicenter, double-blind, randomized clinical trial	European Heart Journal
16	Tse et al. (2007)	Prospective randomized trial of direct endomyocardial implantation of bone marrow cells for treatment of severe coronary artery diseases (PROTECT-CAD trial)	European Heart Journal
17	Cannon et al. (2015)	Efficacy and safety of alirocumab in high cardiovascular risk patients with inadequately controlled hypercholesterolaemia on maximally tolerated doses of statins: the ODYSSEY COMBO II randomized controlled trial	European Heart Journal
18	McNab et al. (2006)	An open label, single-centre, randomized trial of spinal cord stimulation vs. percutaneous myocardial laser revascularization in patients with refractory angina pectoris: the SPiRiT trial	European Heart Journal
19	Hamshere et al. (2015)	Randomized trial of combination cytokine and adult autologous bone marrow progenitor cell administration in patients with non-ischaemic dilated cardiomyopathy: the REGENERATE-DCM clinical trial	European Heart Journal
20	Werner et al. (2018)	A randomized multicentre trial to compare revascularization with optimal medical therapy for the treatment of chronic total coronary occlusions	European Heart Journal
21	Solari et al. (2017)	Stop vasodepressor drugs in reflex syncope: a randomised controlled trial	Heart
22	Goodacre et al. (2011)	The randomised assessment of treatment using panel assay of cardiac markers (RATPAC) trial: a randomised controlled trial of point-of-care cardiac markers in the emergency department	Heart
23	Conway et al. (2018)	Forced air warming during sedation in the cardiac catheterisation laboratory: a randomised controlled trial	Heart
24	Noad et al. (2016)	Beneficial effect of a polyphenol-rich diet on cardiovascular risk: a randomised control trial	Heart
25	Broch et al. (2016)	Controlled release metoprolol for aortic regurgitation: a randomised clinical trial	Heart
26	Palm et al. (2019)	Sexual rehabilitation for cardiac patients with erectile dysfunction: a randomised clinical trial	Heart
27	Meurin et al. (2015)	Colchicine for postoperative pericardial effusion: a multicentre, double-blind, randomised controlled trial	Heart
28	Moretti et al. (2018)	The EUROpean and Chinese cardiac and renal Remote Ischemic Preconditioning Study (EURO-CRIPS CardioGroup I): A randomized controlled trial	International Journal of Cardiology
29	Klausen et al. (2016)	Effects of eHealth physical activity encouragement in adolescents with complex congenital heart disease: The PReVaiL randomized clinical trial	International Journal of Cardiology
30	Carney et al. (2016)	Collaborative care for depression symptoms in an outpatient cardiology setting: a randomized clinical trial	International Journal of Cardiology
31	Miyoshi et al. (2017)	Effect of remote ischemia or nicorandil on myocardial injury following percutaneous coronary intervention in patients with stable coronary artery disease: a randomized controlled trial	International Journal of Cardiology
32	Lichtblau et al. (2019)	Dexamethasone improves pulmonary hemodynamics in COPD-patients going to altitude: A randomized trial	International Journal of Cardiology
33	Viszlayová et al. (2018)	Sonolysis in risk reduction of symptomatic and silent brain infarctions during coronary stenting (SONOREDUCE): randomized, controlled trial	International Journal of Cardiology
34	Rao et al. (2014)	A registry-based randomized trial comparing radial and femoral approaches in women undergoing percutaneous coronary intervention: the SAFE-PCI for Women (Study of Access Site for Enhancement of PCI for Women) trial	JACC-Cardiovascular Intervention
35	Kufner et al. (2016)	Randomized trial of polymer-free sirolimus- and probucol-eluting stents versus durable polymer zotarolimus-eluting stents: 5-year results of the ISAR-TEST-5 trial	JACC-Cardiovascular Intervention
36	Tapson et al. (2018)	A randomized trial of the optimum duration of acoustic pulse thrombolysis procedure in acute intermediate-risk pulmonary embolism: the OPTALYSE PE trial	JACC-Cardiovascular Intervention
37	Takagi et al. (2009)	A prospective, multicenter, randomized trial to assess efficacy of pioglitazone on in-stent neointimal suppression in type 2 diabetes: POPPS (prevention of in-stent neointimal proliferation by pioglitazone study)	JACC-Cardiovascular Intervention
38	Mashayekhi et al. (2018)	Randomized trial to assess regional left ventricular function after stent implantation in chronic total occlusion: the REVASC trial	JACC-Cardiovascular Intervention
39	Han et al. (2018)	A Randomized Trial Comparing the NeoVas Sirolimus-Eluting Bioresorbable Scaffold and Metallic Everolimus-Eluting Stents	JACC-Cardiovascular Intervention
40	Di et al. (2009)	The PASEO (PaclitAxel or sirolimus-eluting stent versus bare metal stent in primary angioplasty) randomized trial	JACC-Cardiovascular Intervention
41	Antoniucci et al. (2003)	A randomized trial comparing primary infarct artery stenting with or without abciximab in acute myocardial infarction	Journal of the American College of Cardiology
42	Jones et al. (2013)	A randomized trial to assess catheter ablation versus rate control in the management of persistent atrial fibrillation in heart failure	Journal of the American College of Cardiology
43	Carrick et al. (2014)	A randomized trial of deferred stenting versus immediate stenting to prevent no- or slow-reflow in acute ST-segment elevation myocardial infarction (DEFER-STEMI)	Journal of the American College of Cardiology
44	Carrié et al. (2012)	A multicenter randomized trial comparing amphilimus- with paclitaxel-eluting stents in de novo native coronary artery lesions	Journal of the American College of Cardiology
45	Alfonso et al. (2015)	A prospective randomized trial of drug-eluting balloons versus everolimus-eluting stents in patients with in-stent restenosis of drug-eluting stents: the RIBS IV randomized clinical trial	Journal of the American College of Cardiology
46	Chen et al. (2017)	Double Kissing Crush Versus Provisional Stenting for Left Main Distal Bifurcation Lesions: DKCRUSH-V Randomized Trial	Journal of the American College of Cardiology
47	Gershlick et al. (2015)	Randomized trial of complete versus lesion-only revascularization in patients undergoing primary percutaneous coronary intervention for STEMI and multivessel disease: the CvLPRIT trial	Journal of the American College of Cardiology

© 2020 Wang F et al. *JAMA Network Open.*

eAppendix. Step-by-Step User Guide

CONSORT-NLP User Guide (Version 1.2)

Before you start

Here are a few things to know before you start.

- CONSORT-NLP is an artificial intelligence tool to automatically generate the reporting checklist for parallel group randomized trials.
- The accepted document format for CONSORT-NLP is the widely used Portable Document Format (PDF).

To learn more about how to generate PDF from Word Document, check out Prepare Input Document. Or you can jump to Getting Started with CONSORT-NLP.

For more details, you can visit www.consort-nlp.org.

© 2020 Wang F et al. *JAMA Network Open.*

Prepare Input Document

The accepted document format for the tool is the widely used Portable Document Format (PDF). You can use Microsoft Word to convert Word Processing Document (.doc or .docx) to pdf.

Here, we use Microsoft Word 2016 to create a PDF document.

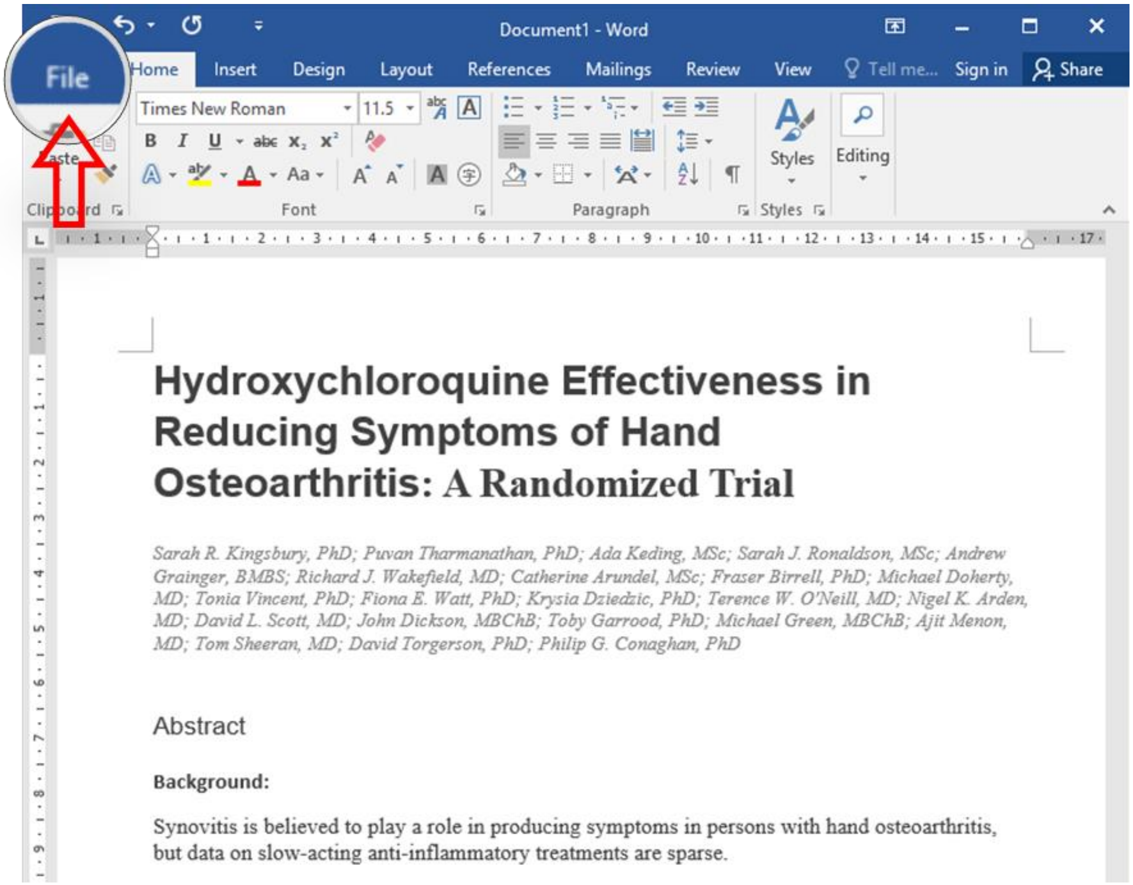

First, **click** **File** in the left corner of the menu bar.

© 2020 Wang F et al. *JAMA Network Open.*

Doing so opens a pop-out window.

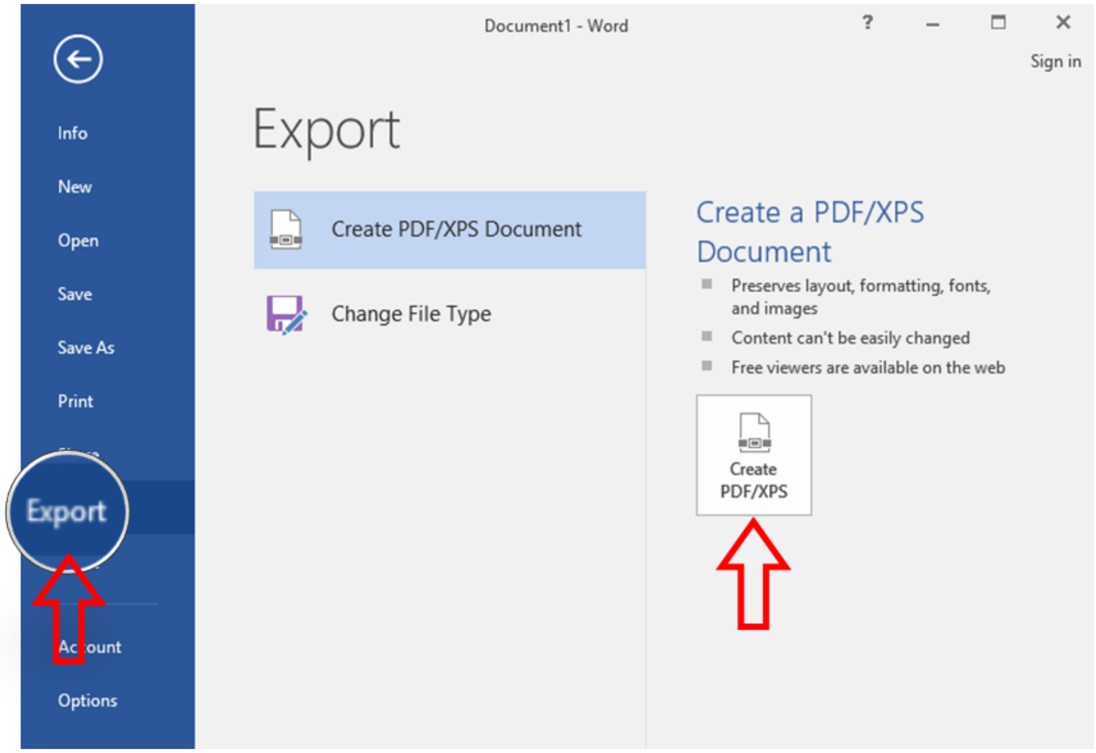

Click **Export** on the left column of options and **click** **Create PDF/XPS** button on the right side to generate a pdf file.

You can save the created PDF under your desired filename and a directory of your choice.

© 2020 Wang F et al. *JAMA Network Open.*

Getting Started with CONSORT-NLP

1. Launch CONSORT-NLP

- Windows OS Users:

  Double-click on the ‘CONSORT-NLP_v1.0’ file to start CONSORT-NLP.

- Linux OS Users:

  Call CONSORT-NLP_v1.0 through terminal with the following command:

```
cd /path/to/CONSORT-NLP
java -jar CONSORT-NLP_v1.0.jar
```

- Mac OS Users:
  - First Use:

    Right-click the ‘CONSORT-NLP_v1.0’ file for the first use.

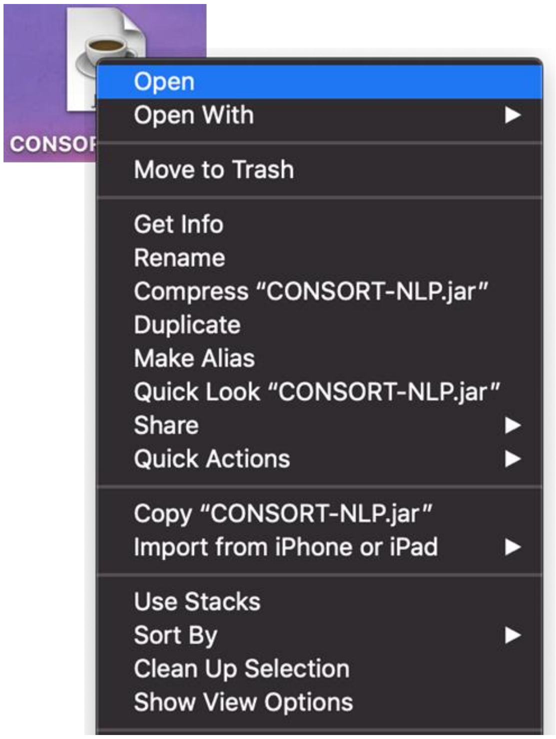

Click Open in the pop-up menu.

© 2020 Wang F et al. *JAMA Network Open.*

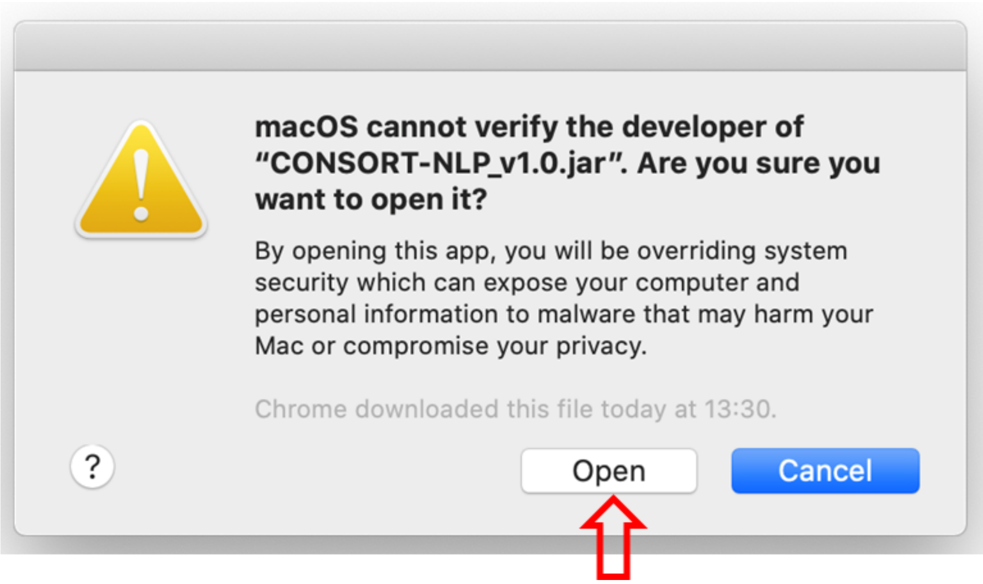

Click the **Open** button in the pop-up warning dialogue.

- Subsequent Sessions:

  After the first launch, CONSORT-NLP can be started simply by **double-clicking** the icon.

© 2020 Wang F et al. *JAMA Network Open.*

2. Input document

There are two options for document input.

- Option 1: **click** the **Browse** button to select the document you want to input OR directly enter the path of a file in the text box.
- Option 2: **drag** the file to the drag and drop area.

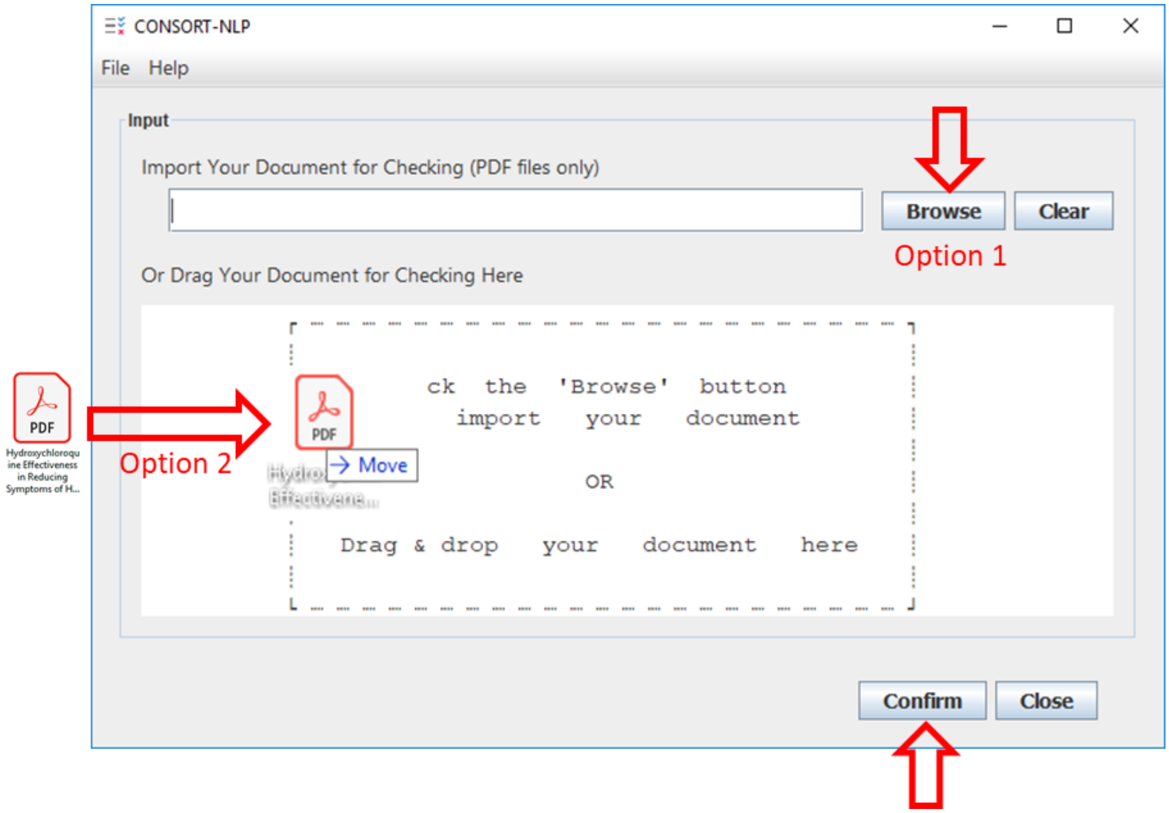

Click the **Confirm** button after uploading the document.

© 2020 Wang F et al. *JAMA Network Open.*

3. A ‘Please Wait…’ pop-up will appear

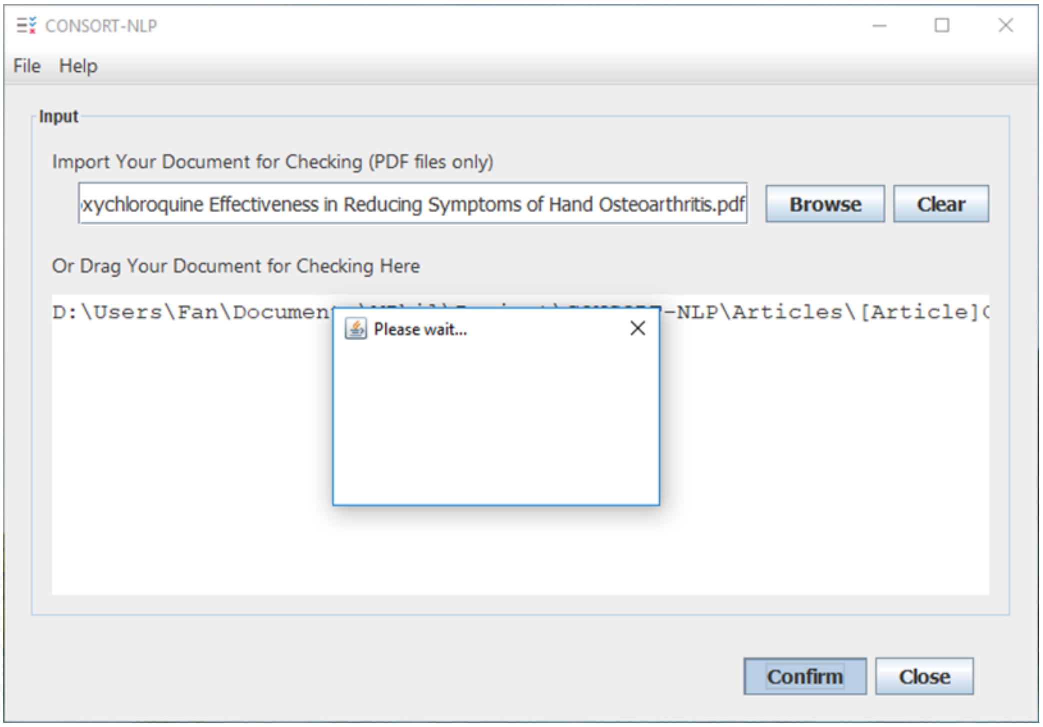

After around 10-20 seconds, CONSORT-NLP will turn to the next page.

- For Mac OS Users:

  If the following warning dialogue appears:

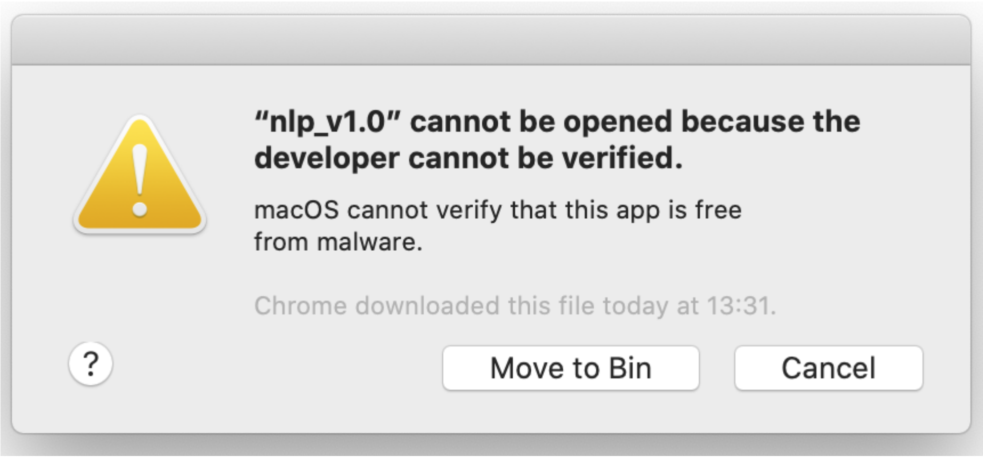

  Open System Preferences and click Security & Privacy. Change "Allow apps downloaded from" to Anywhere. Once done, try Step2 again.

© 2020 Wang F et al. *JAMA Network Open.*

The second page of CONSORT-NLP mainly includes three parts: a. Article view, b. Matching sentence view, c. Checklist items.

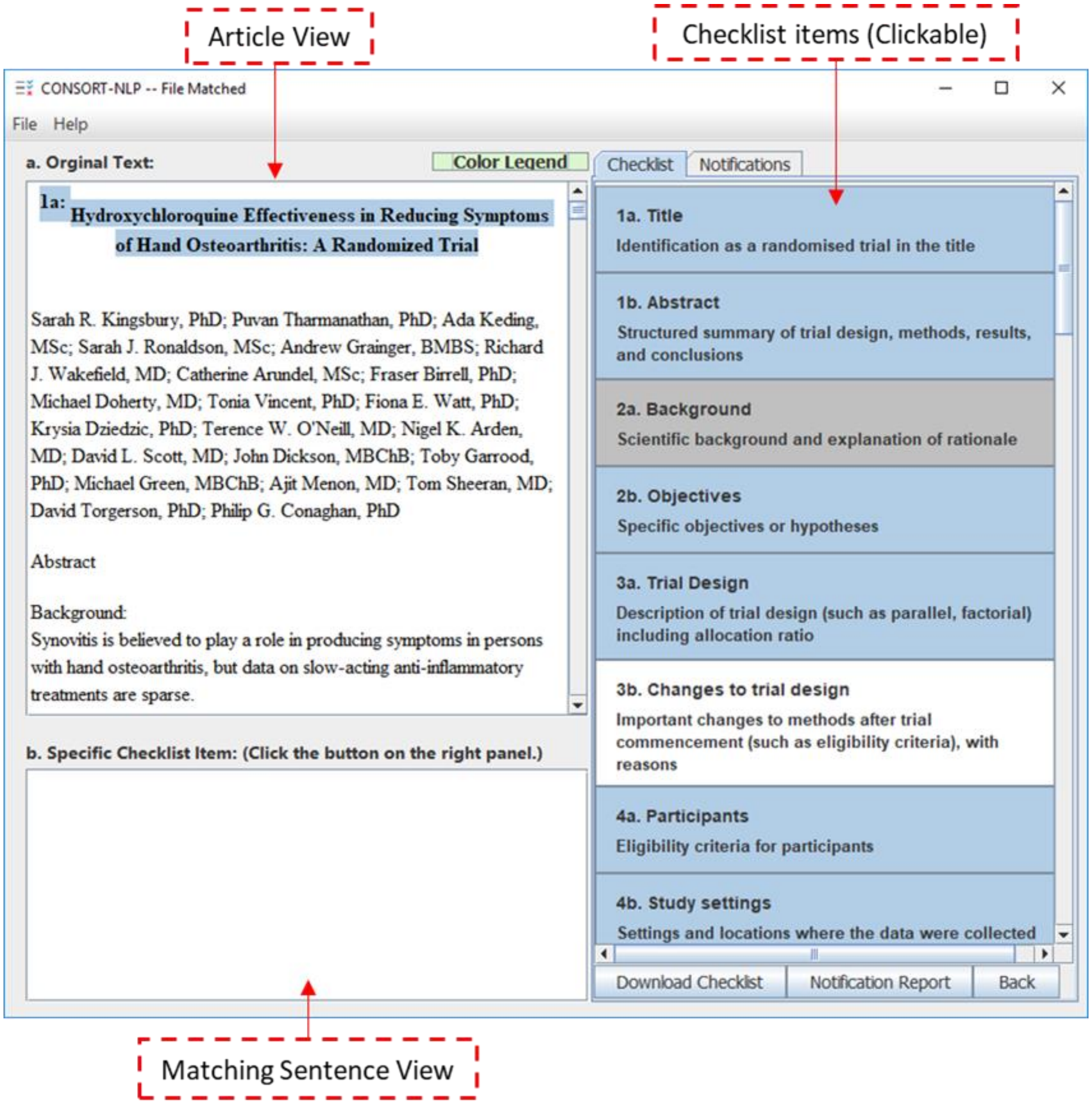

© 2020 Wang F et al. *JAMA Network Open.*

Clickable checklist items are colored differently.

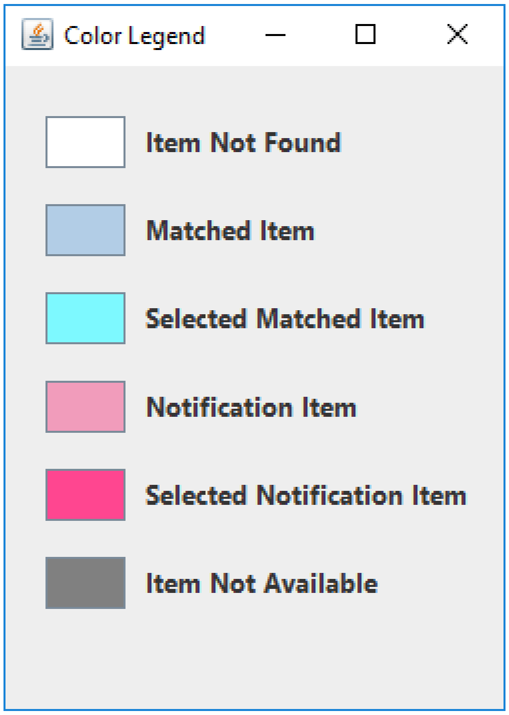

Click the **Color Legend** button to get the meaning of these colors.

© 2020 Wang F et al. *JAMA Network Open.*

4. Use CONSORT-NLP

Click on a checklist item button, the detected sentences will be shown in the Matching Sentence View.

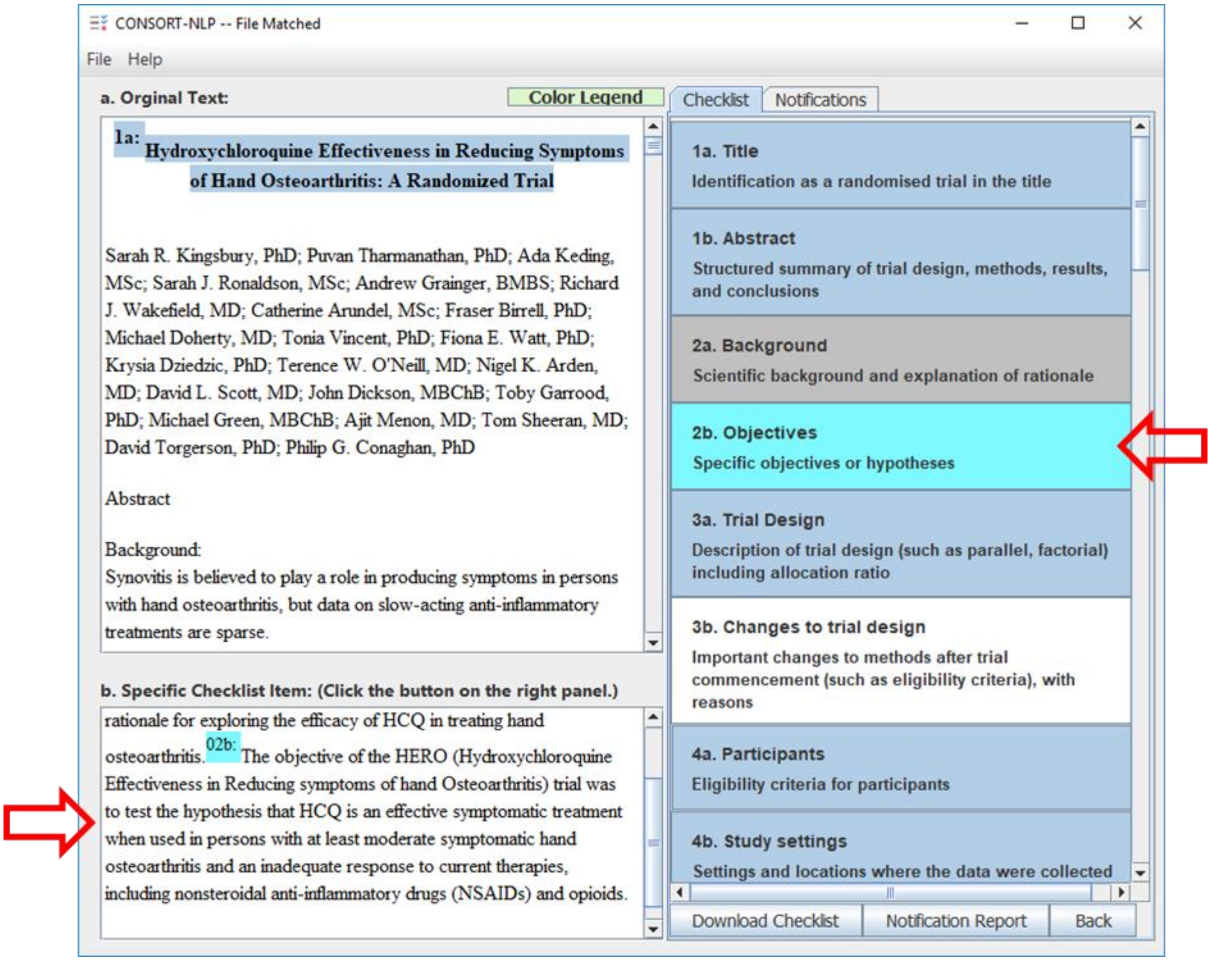

For example, here we **clicked** the button **2b. Objectives**. The detected objectives for this study would be shown.

© 2020 Wang F et al. *JAMA Network Open.*

You can also **scroll down** the original text in Article View to find the matched sentence to each item. The matched sentences are highlighted according to the color legend. The sentence prefix indicates the item number of the relevant CONSORT item.

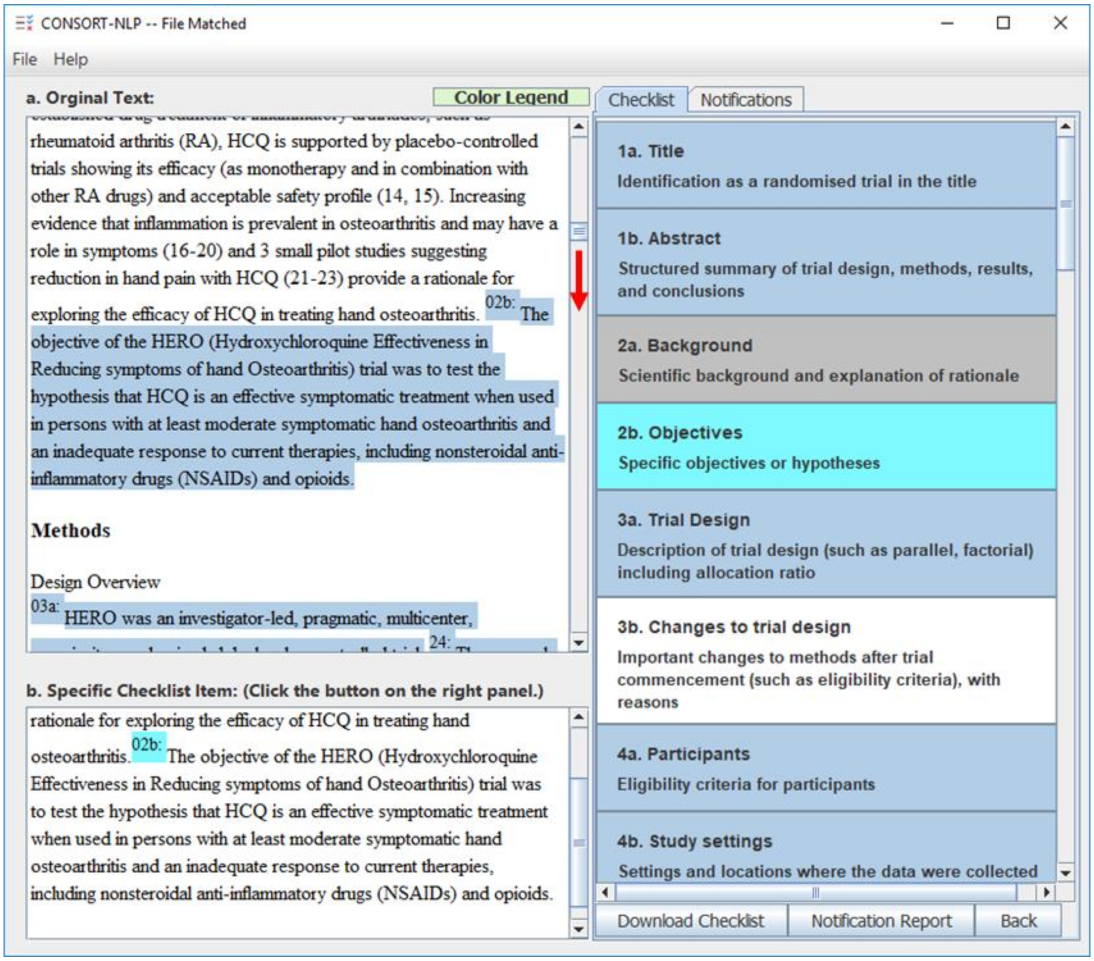

© 2020 Wang F et al. *JAMA Network Open.*

Click on a notification item in the checklist view, the matching sentence view will display the notification message for this item.

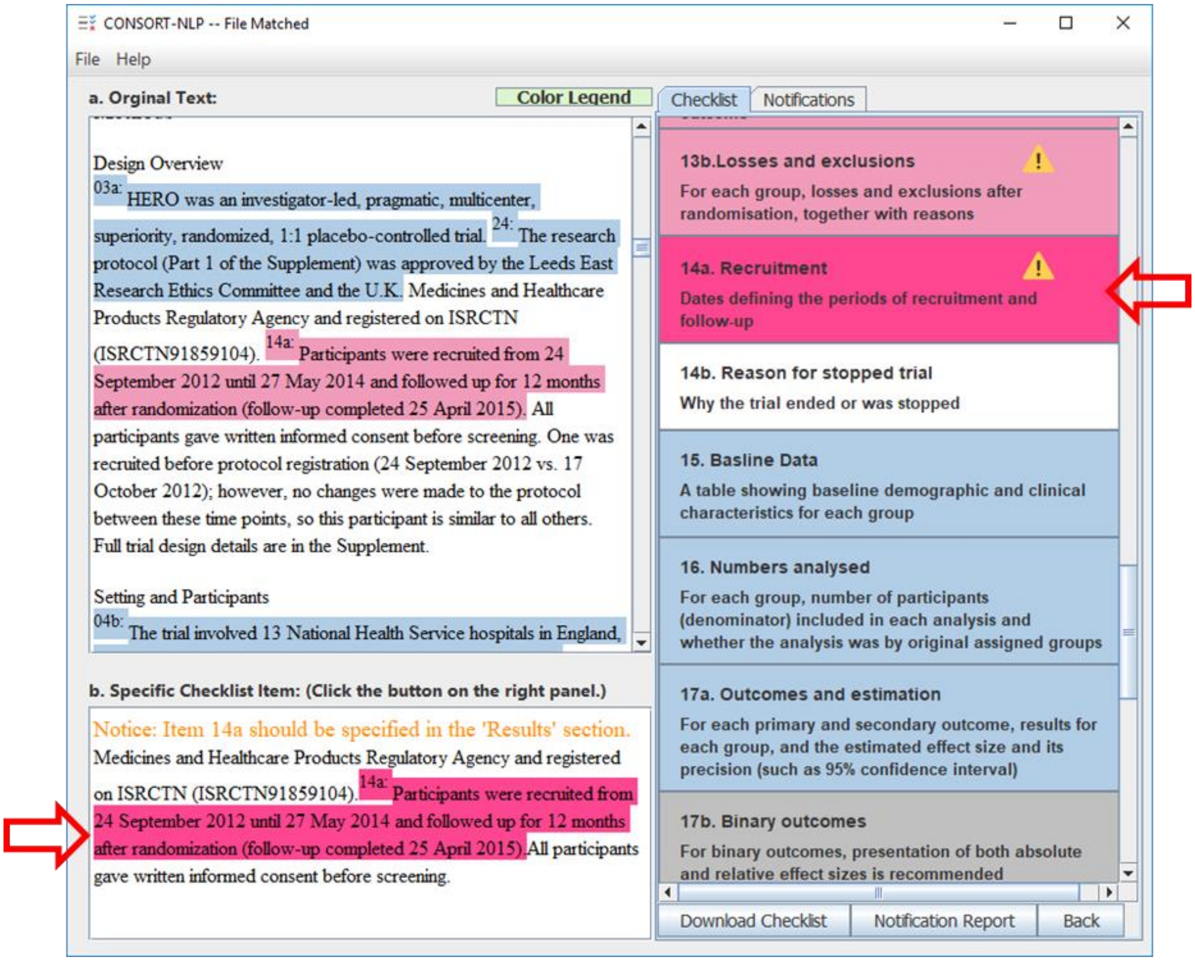

According to the CONSORT list, dates defining the periods of recruitment and enrollment should be specified in the 'Results' section. However, in this article, this information only appears in the 'Methods' part in the main content.

© 2020 Wang F et al. *JAMA Network Open.*

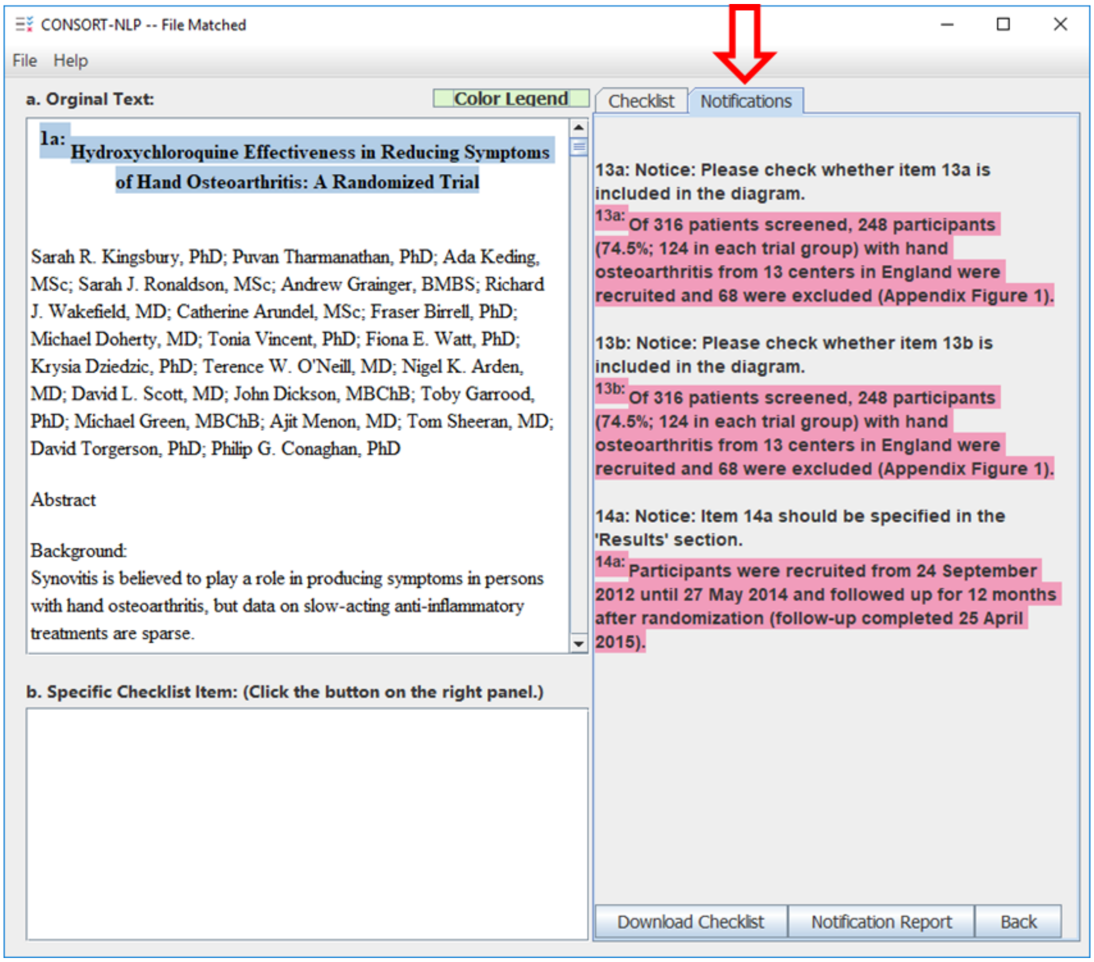

Click on the **Notifications** tab, it will turn to a new panel which displays list of notification items.

© 2020 Wang F et al. *JAMA Network Open.*

5. Export checklist and notification report

CONSORT-NLP can generate a CONSORT checklist and a notification report summarizing all the notification items.

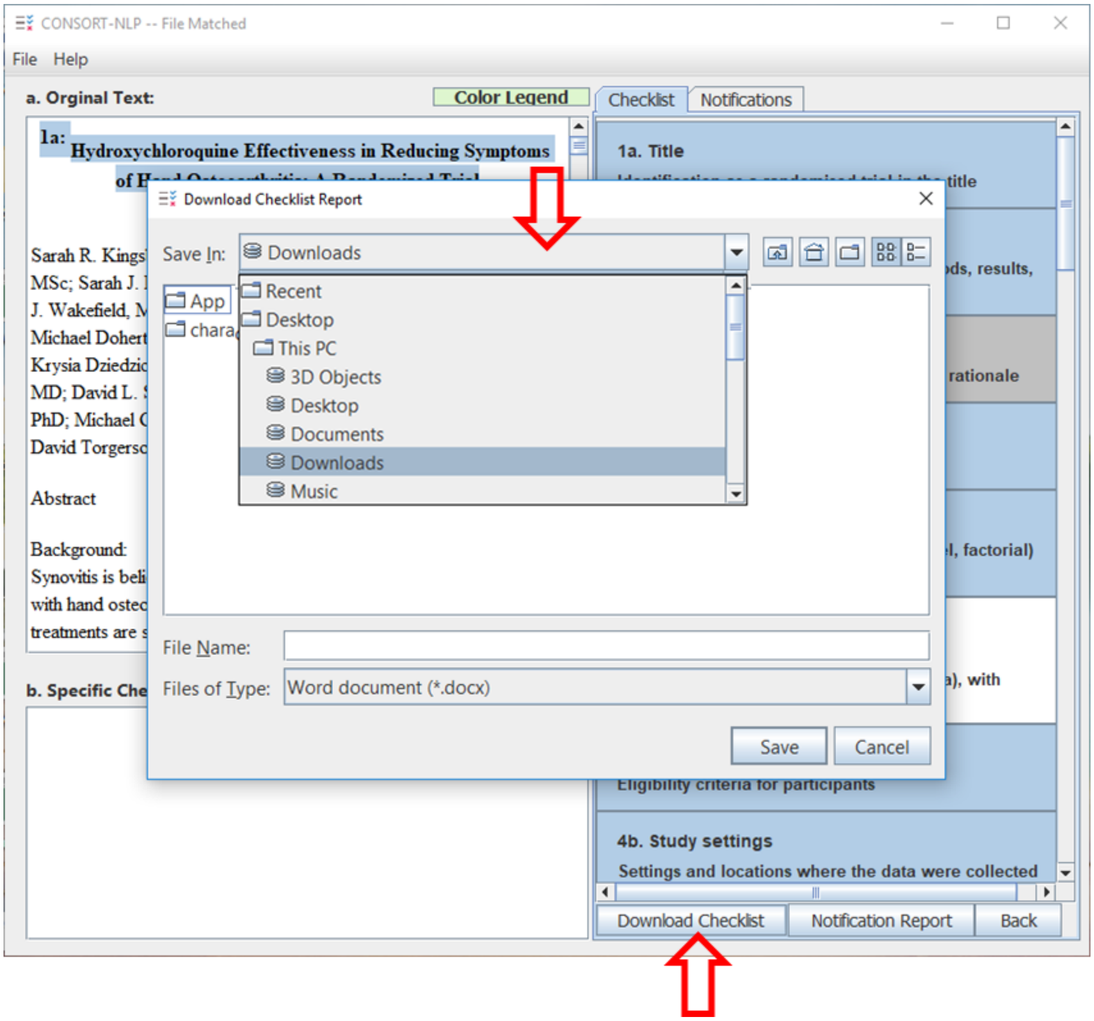

Click the **Download Checklist** button, a pop-up window will appear. You can save the CONSORT Checklist under your desired filename and a directory of your choice.

© 2020 Wang F et al. *JAMA Network Open.*

The CONSORT checklist is saved as a Word Document (.docx). You can open the file and modify the document if needed.

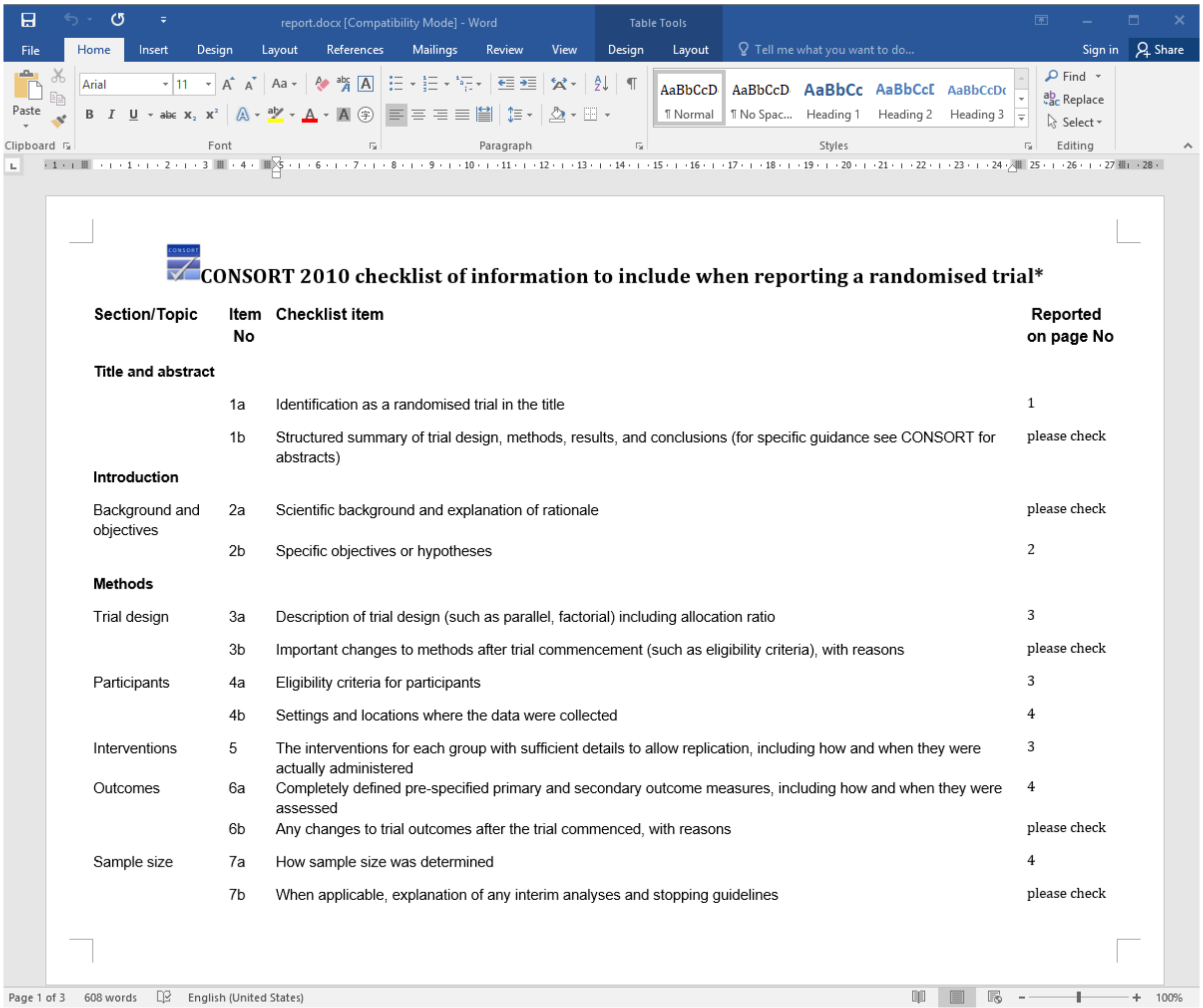

CONSORT 2010 checklist of information to include when reporting a randomised trial*

Section/Topic	Item No	Checklist item	Reported on page No
**Title and abstract**			
	1a	Identification as a randomised trial in the title	1
	1b	Structured summary of trial design, methods, results, and conclusions (for specific guidance see CONSORT for abstracts)	please check
**Introduction**			
Background and objectives	2a	Scientific background and explanation of rationale	please check
	2b	Specific objectives or hypotheses	2
**Methods**			
Trial design	3a	Description of trial design (such as parallel, factorial) including allocation ratio	3
	3b	Important changes to methods after trial commencement (such as eligibility criteria), with reasons	please check
Participants	4a	Eligibility criteria for participants	3
	4b	Settings and locations where the data were collected	4
Interventions	5	The interventions for each group with sufficient details to allow replication, including how and when they were actually administered	3
Outcomes	6a	Completely defined pre-specified primary and secondary outcome measures, including how and when they were assessed	4
	6b	Any changes to trial outcomes after the trial commenced, with reasons	please check
Sample size	7a	How sample size was determined	4
	7b	When applicable, explanation of any interim analyses and stopping guidelines	please check

© 2020 Wang F et al. *JAMA Network Open.*

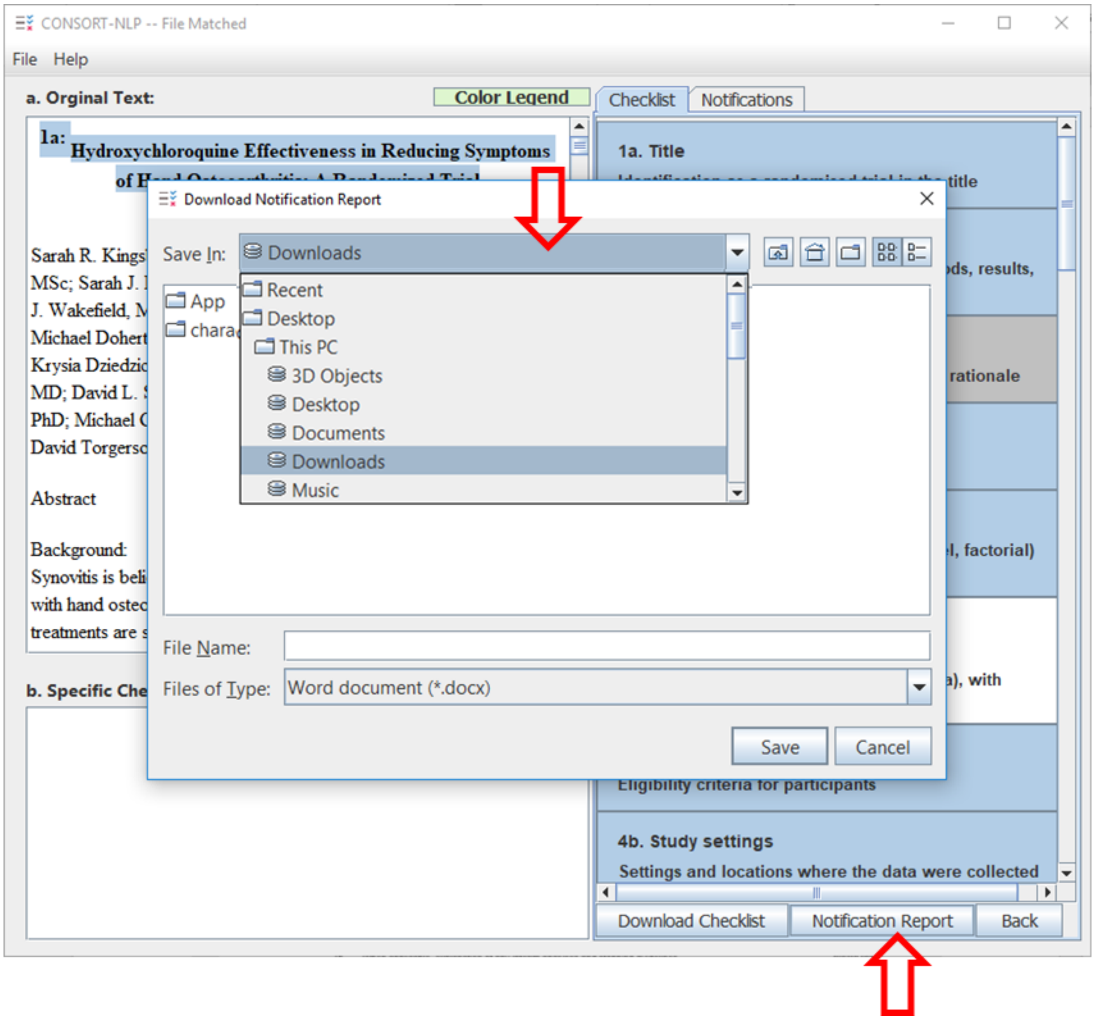

Click on the **Notification Report** button, a pop-up window will appear. You can save the notification report under your desired filename and a directory of your choice.

© 2020 Wang F et al. *JAMA Network Open.*

The notification report will be saved as a Word Document (.docx).

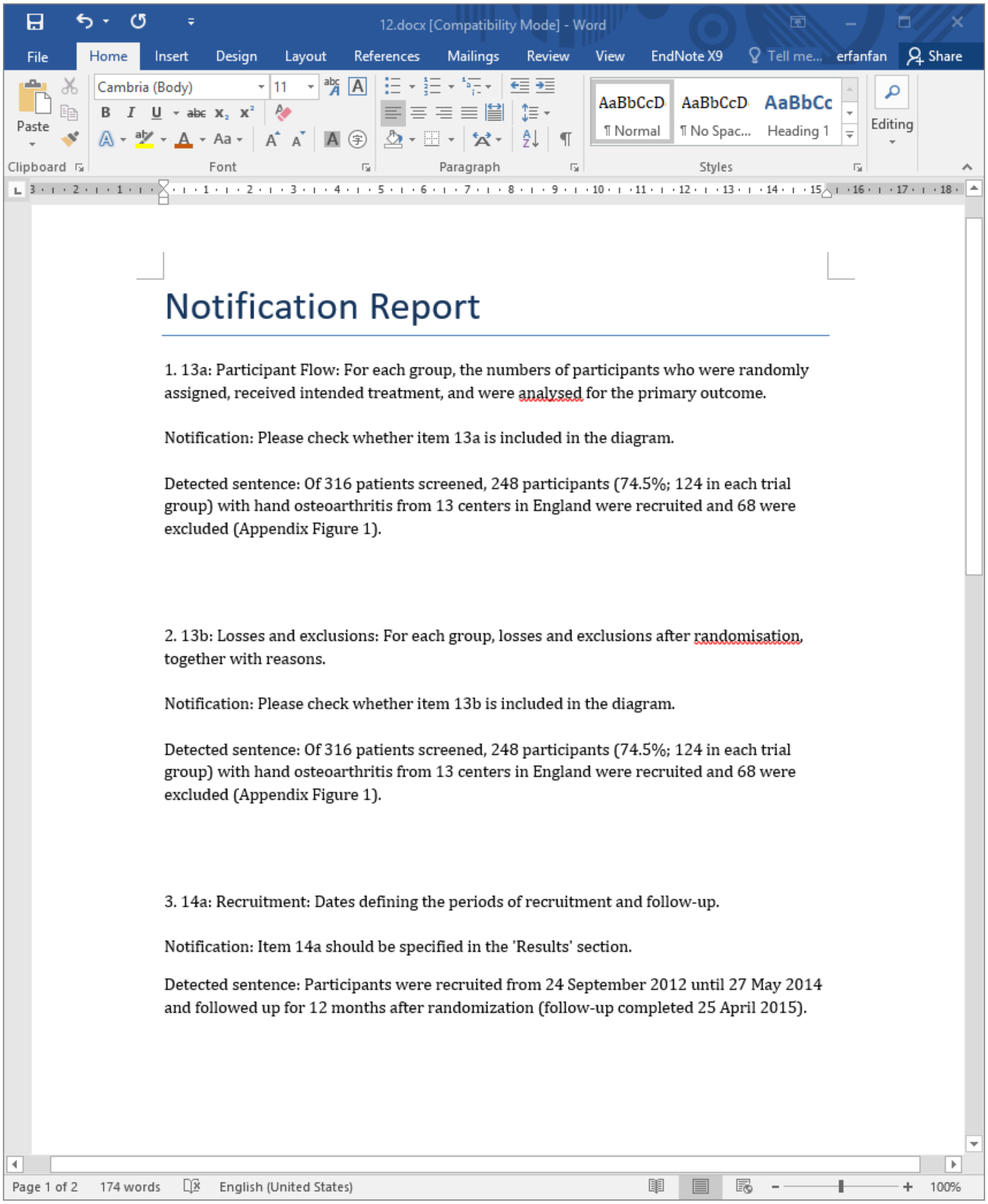

Notification Report

1. 13a: Participant Flow: For each group, the numbers of participants who were randomly assigned, received intended treatment, and were analysed for the primary outcome.

Notification: Please check whether item 13a is included in the diagram.

Detected sentence: Of 316 patients screened, 248 participants (74.5%; 124 in each trial group) with hand osteoarthritis from 13 centers in England were recruited and 68 were excluded (Appendix Figure 1).

2. 13b: Losses and exclusions: For each group, losses and exclusions after randomisation, together with reasons.

Notification: Please check whether item 13b is included in the diagram.

Detected sentence: Of 316 patients screened, 248 participants (74.5%; 124 in each trial group) with hand osteoarthritis from 13 centers in England were recruited and 68 were excluded (Appendix Figure 1).

3. 14a: Recruitment: Dates defining the periods of recruitment and follow-up.

Notification: Item 14a should be specified in the 'Results' section.

Detected sentence: Participants were recruited from 24 September 2012 until 27 May 2014 and followed up for 12 months after randomization (follow-up completed 25 April 2015).

© 2020 Wang F et al. *JAMA Network Open.*

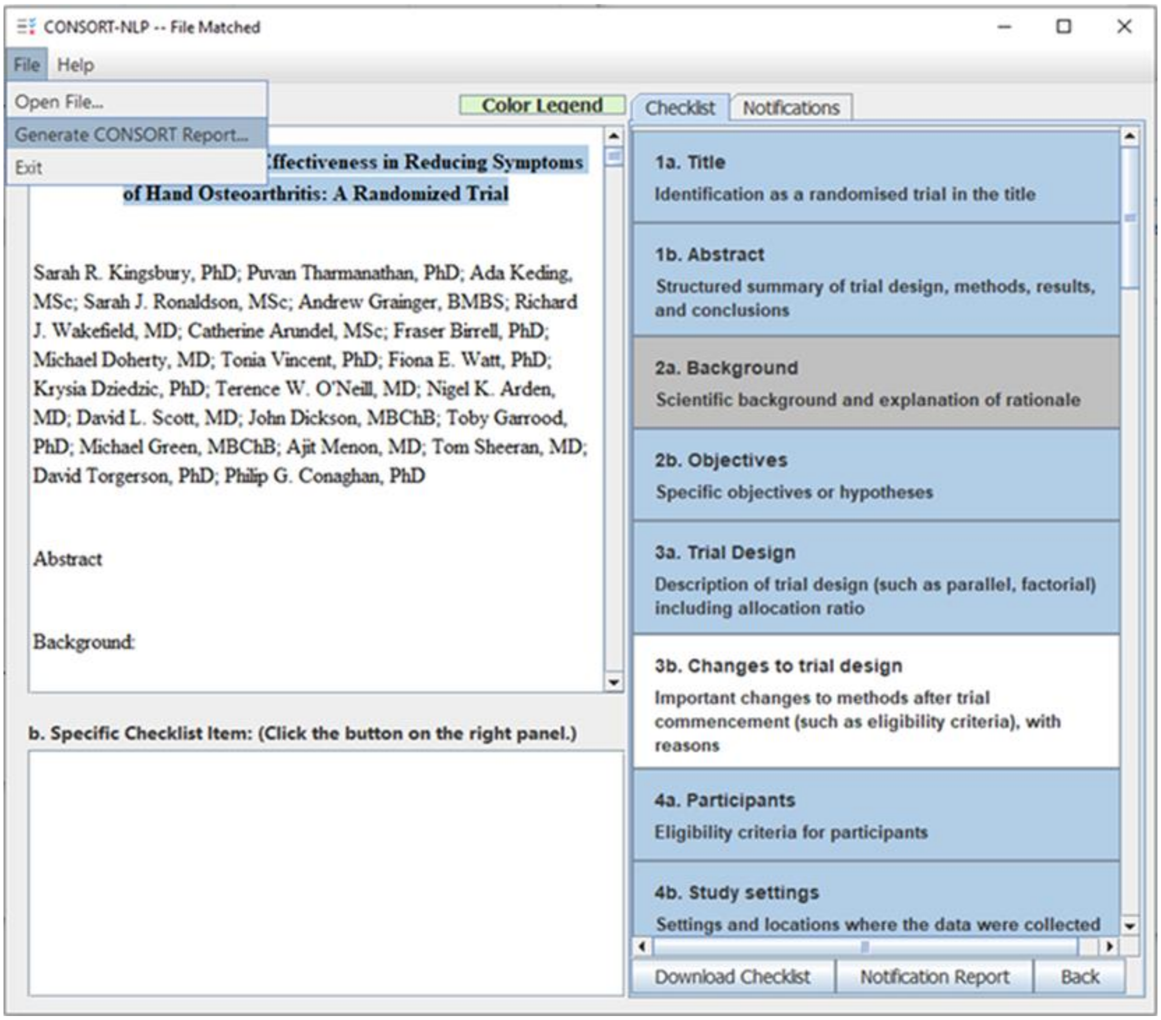

Click on the **Generate CONSORT Report** button listed in the **File** menu, a pop-up window will appear. You can save a detailed report under your desired filename and a directory of your choice.

© 2020 Wang F et al. *JAMA Network Open.*

The detailed report will be saved as a Word Document (.docx).

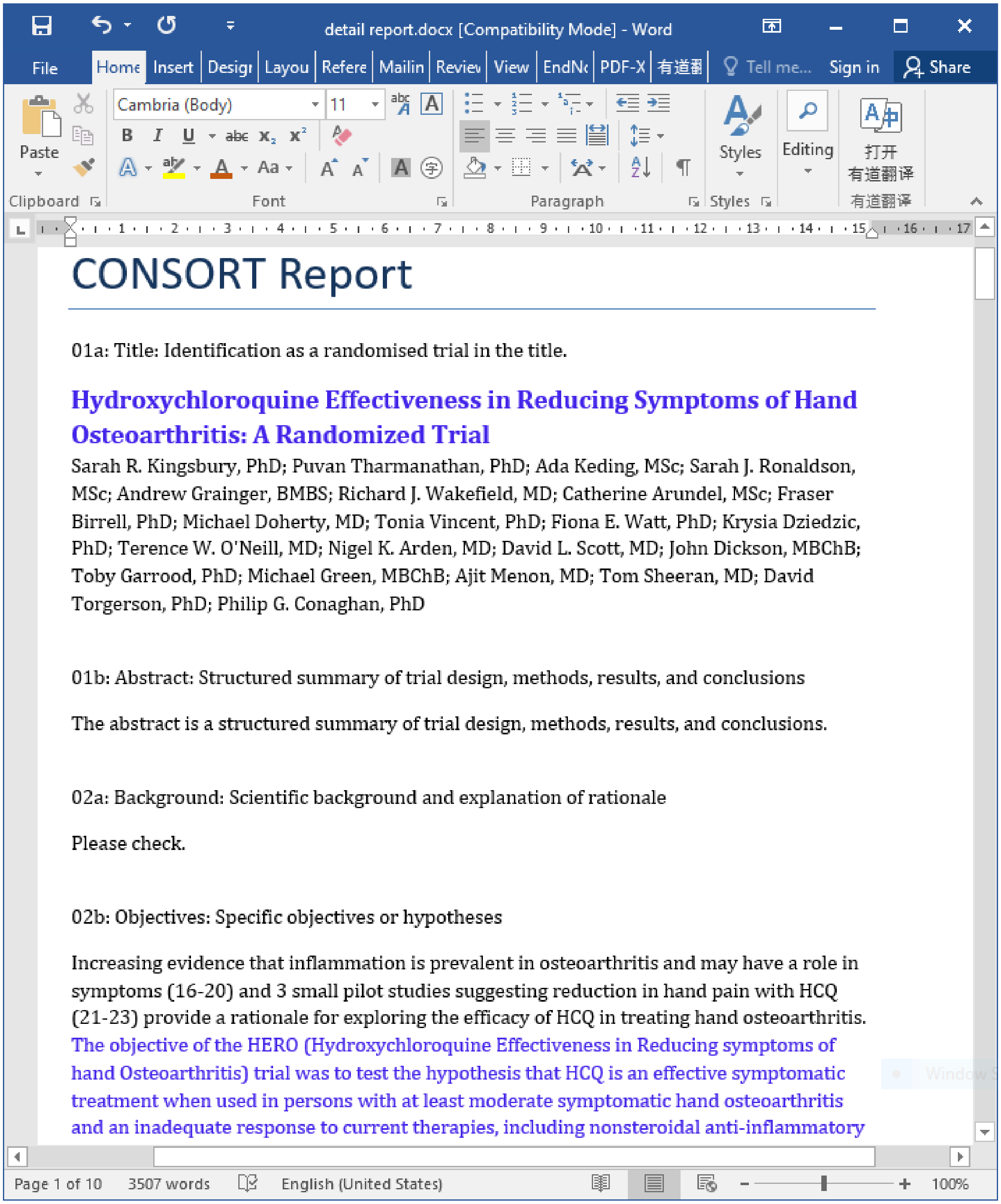

CONSORT Report

01a: Title: Identification as a randomised trial in the title.

Hydroxychloroquine Effectiveness in Reducing Symptoms of Hand Osteoarthritis: A Randomized Trial

Sarah R. Kingsbury, PhD; Puvan Tharmanathan, PhD; Ada Keding, MSc; Sarah J. Ronaldson, MSc; Andrew Grainger, BMBS; Richard J. Wakefield, MD; Catherine Arundel, MSc; Fraser Birrell, PhD; Michael Doherty, MD; Tonia Vincent, PhD; Fiona E. Watt, PhD; Krysia Dziedzic, PhD; Terence W. O'Neill, MD; Nigel K. Arden, MD; David L. Scott, MD; John Dickson, MBChB; Toby Garrood, PhD; Michael Green, MBChB; Ajit Menon, MD; Tom Sheeran, MD; David Torgerson, PhD; Philip G. Conaghan, PhD

01b: Abstract: Structured summary of trial design, methods, results, and conclusions

The abstract is a structured summary of trial design, methods, results, and conclusions.

02a: Background: Scientific background and explanation of rationale

Please check.

02b: Objectives: Specific objectives or hypotheses

Increasing evidence that inflammation is prevalent in osteoarthritis and may have a role in symptoms (16-20) and 3 small pilot studies suggesting reduction in hand pain with HCQ (21-23) provide a rationale for exploring the efficacy of HCQ in treating hand osteoarthritis. The objective of the HERO (Hydroxychloroquine Effectiveness in Reducing symptoms of hand Osteoarthritis) trial was to test the hypothesis that HCQ is an effective symptomatic treatment when used in persons with at least moderate symptomatic hand osteoarthritis and an inadequate response to current therapies, including nonsteroidal anti-inflammatory

© 2020 Wang F et al. *JAMA Network Open.*

6. Back to import page

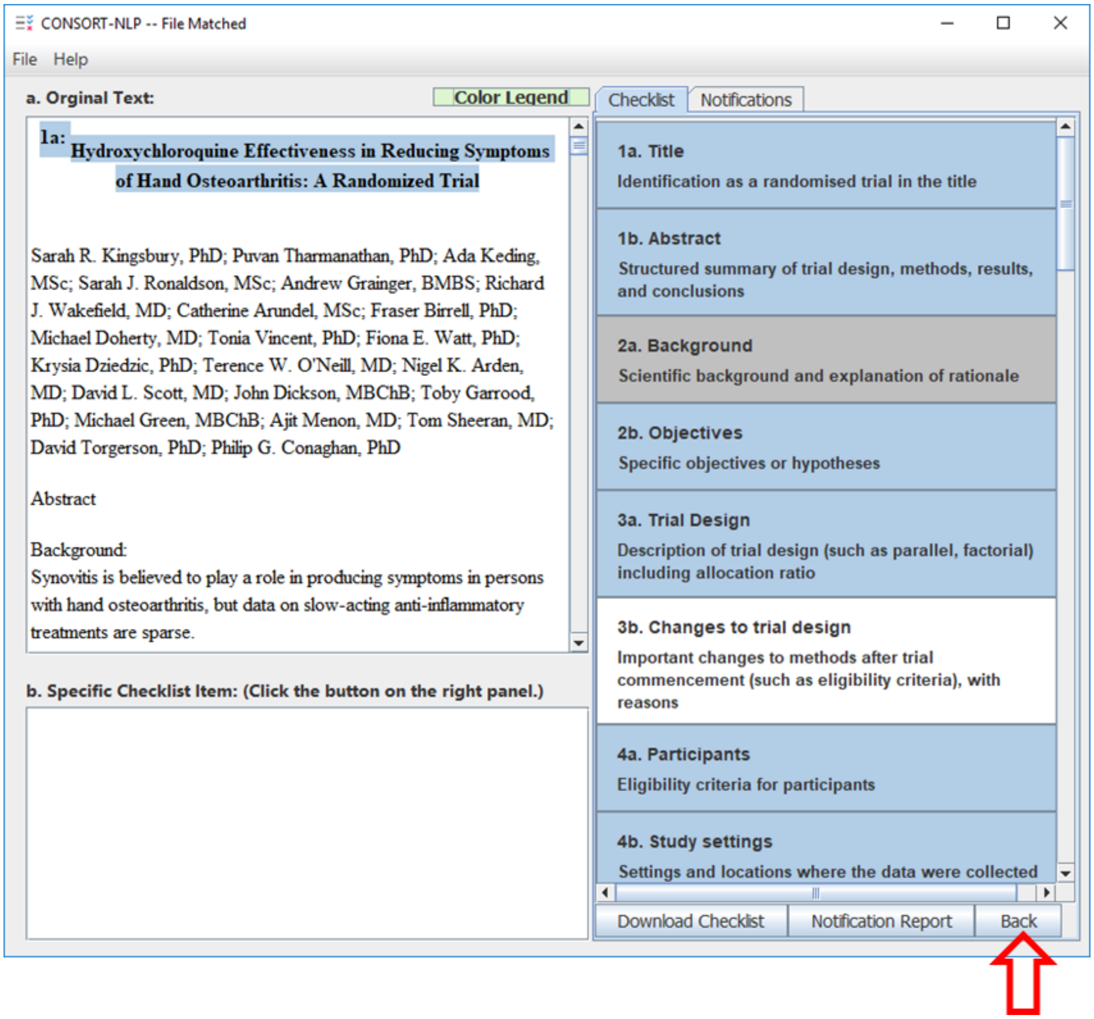

To check another document, **click** the **Back** button to get back to the import page.

© 2020 Wang F et al. *JAMA Network Open.*

7. Exit CONSORT-NLP

There are two ways to close CONSORT-NLP.

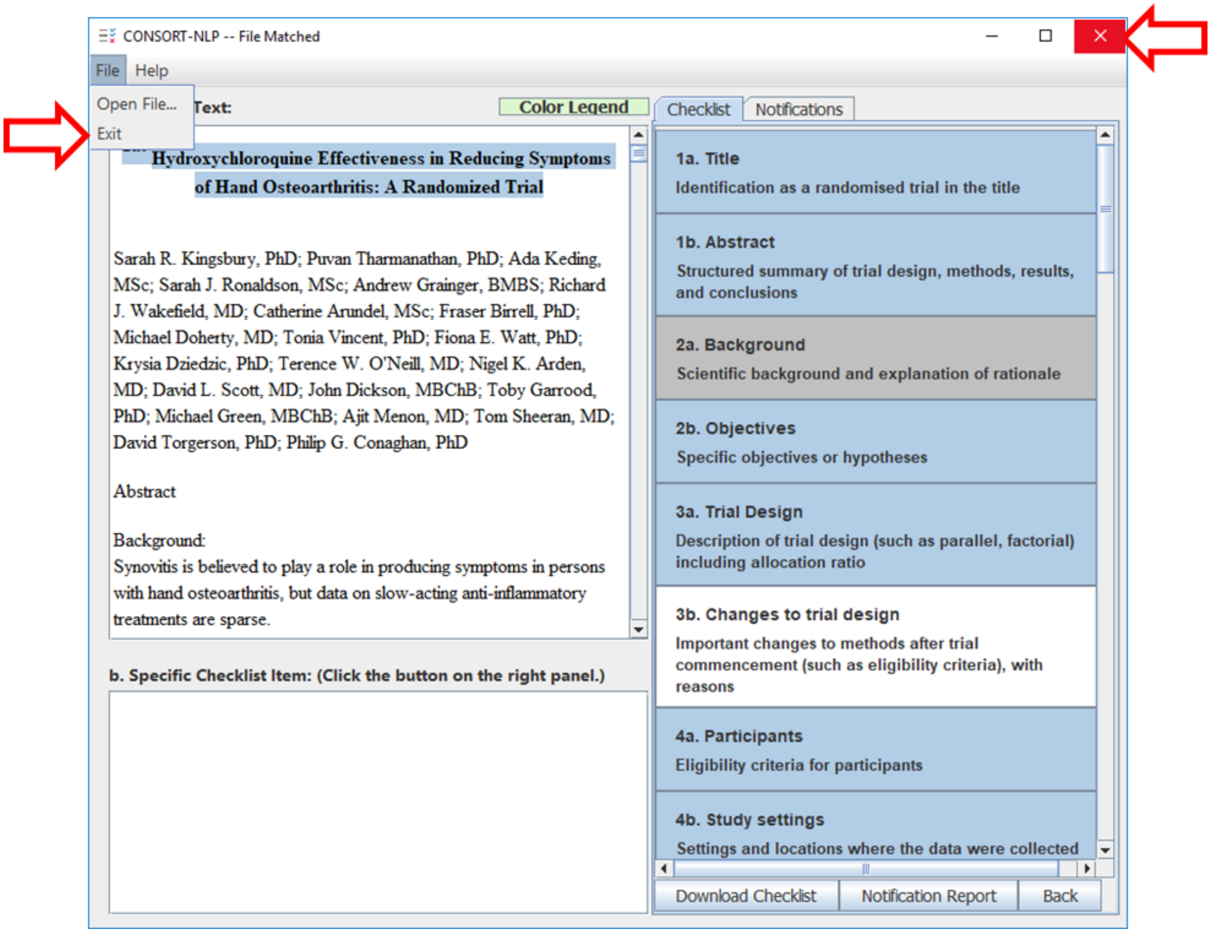

You can **click** the **X** in the top-right corner of the window or **click** the **Exit** button listed in the **File** menu.

© 2020 Wang F et al. *JAMA Network Open.*
